# A non-canonical nucleophile unlocks a new mechanistic pathway in a designed enzyme

**DOI:** 10.1038/s41467-024-46123-z

**Published:** 2024-03-04

**Authors:** Amy E. Hutton, Jake Foster, Rebecca Crawshaw, Florence J. Hardy, Linus O. Johannissen, Thomas M. Lister, Emilie F. Gérard, Zachary Birch-Price, Richard Obexer, Sam Hay, Anthony P. Green

**Affiliations:** Manchester Institute of Biotechnology, School of Chemistry, The University of Manchester, Manchester, UK

**Keywords:** Enzymes, Enzyme mechanisms, Biocatalysis

## Abstract

Directed evolution of computationally designed enzymes has provided new insights into the emergence of sophisticated catalytic sites in proteins. In this regard, we have recently shown that a histidine nucleophile and a flexible arginine can work in synergy to accelerate the Morita-Baylis-Hillman (MBH) reaction with unrivalled efficiency. Here, we show that replacing the catalytic histidine with a non-canonical *N*_δ_-methylhistidine (MeHis23) nucleophile leads to a substantially altered evolutionary outcome in which the catalytic Arg124 has been abandoned. Instead, Glu26 has emerged, which mediates a rate-limiting proton transfer step to deliver an enzyme (BH_MeHis_1.8) that is more than an order of magnitude more active than our earlier MBHase. Interestingly, although MeHis23 to His substitution in BH_MeHis_1.8 reduces activity by 4-fold, the resulting His containing variant is still a potent MBH biocatalyst. However, analysis of the BH_MeHis_1.8 evolutionary trajectory reveals that the MeHis nucleophile was crucial in the early stages of engineering to unlock the new mechanistic pathway. This study demonstrates how even subtle perturbations to key catalytic elements of designed enzymes can lead to vastly different evolutionary outcomes, resulting in new mechanistic solutions to complex chemical transformations.

## Abstract (web summary)

The authors previously showed that a histidine nucleophile and a flexible arginine can work in synergy to accelerate the Morita Baylis-Hillman (MBH) reaction. Here, they report another efficient MBHase that employs a non-canonical *N*_δ_-methylhistidine nucleophile paired with a catalytic glutamate, providing an alternative mechanistic solution for MBH catalysis.

## Introduction

Computational enzyme design offers exciting opportunities to develop enzymes with catalytic mechanisms and functions that are beyond those found in nature^1,2^. Powerful programs such as ORBIT^3^, RosettaMatch and RosettaDesign^2,4,5^ have allowed the design of protein catalysts for a range of transformations, including Diels-Alder cycloadditions^6^, Kemp eliminations^7,8^, and retro-aldol reactions^9^. Although the efficiencies of these designs have been relatively low, they can be optimized through directed evolution to generate proficient catalysts, in some cases with efficiencies approaching natural enzymes^10–14^. Recently, our lab and others have shown how an expanded genetic code can be used to broaden the range of catalytic mechanisms that can be embedded into proteins^15–20^. Of particular note, we have shown that the non-canonical amino acid *N*_δ_-methylhistidine (MeHis) can serve as a competent catalytic nucleophile for the development of de novo hydrolases^20^. Although there are some similarities between imidazole and methyl imidazole side chains (for example they have similar p*K*_a_ values of 7.2 and 7.4, respectively^21^), there are also clear differences in their molecular features that can impact their reactivity and/or optimal positioning within protein active sites. Where MeHis exists as a single tautomer, histidine can exist in two tautomeric forms, the partitioning of which is controlled by the protein environment. Catalytic histidines are most commonly activated by hydrogen bonding to the non-reacting nitrogen, whereas these interactions are not available with MeHis meaning that other interactions are likely required for its activation as a catalytic nucleophile. Finally, with MeHis, catalytic intermediates unambiguously exist as charged imidazolium ions, whereas with His, multiple states can exist as a result of deprotonation/protonation of the non-coordinating nitrogen. The potential impact of such species on catalytic mechanisms is clearly demonstrated in our earlier work on artificial hydrolase engineering, whereby histidine methylation prevented the formation of unreactive acyl-enzyme intermediates that compromise the activity of designed hydrolases equipped with canonical nucleophiles^20,22,23^. In this way, MeHis can be considered a genetically encodable surrogate of the widely employed nucleophilic catalyst DMAP^24^.

**Fig. 1:**
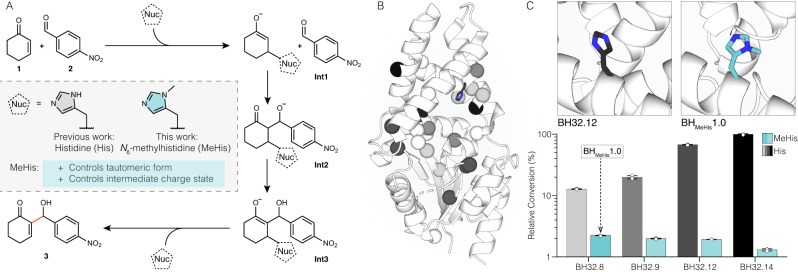
Investigation of the utility of MeHis as a nucleophile for the MBH reaction and identification of a suitable starting point for directed evolution. **A** Chemical scheme for the MBH reaction between 2-cyclohexen-1-one (**1**) and 4-nitrobenzaldehyde (**2**) to form MBH product 2-(hydroxy(4-nitrophenyl)methyl)cyclohex-2-en-1-one (**3**). Previous work has afforded the MBHase BH32.14^13^ which promotes catalysis *via* a histidine nucleophile. In this work, His23 is replaced by *N*_δ_-methylhistidine (MeHis) for the creation of a more efficient MBHase. Nuc = nucleophile. **B** Crystal structure of BH32.12 (PDB: 6Z1L, https://www.rcsb.org/structure/6Z1L) showing the positions of the amino acids mutated during BH32.14 evolution (represented as spheres at the C_α_). Mutations are shaded in grey scale according to their order of introduction corresponding to the evolutionary trajectory shown in Fig. 1C. His23 is shown as atom-coloured sticks with black carbon atoms. **C** The active sites of BH32.12 (PDB: 6Z1L, https://www.rcsb.org/structure/6Z1L) and BH_MeHis_1.0 (PDB: 8BP1, https://www.rcsb.org/structure/8BP1) are shown with His23 and MeHis23 nucleophiles shown as atom-coloured sticks with black and blue carbon atoms, respectively. Comparisons of activity of BH32.14 and selected variants along the BH32 evolutionary trajectory with either His (grey scale) or MeHis (blue) as the catalytic nucleophile at position 23. BH32.8 His23MeHis (subsequently referred to as BH_MeHis_1.0) was selected for further engineering. Biotransformations were performed using **1** (15 mM), **2** (1.5 mM) and enzyme (60 µM) in PBS (pH 7.4) with 3% (v/v) MeCN as cosolvent and analyzed by ultra-high performance liquid chromatography (UPLC) following 5 h incubation at 30 °C. Error bars represent the standard deviation of measurements made in triplicate centred around the averaged value. Source data are provided as a Source Data file.

In light of its favourable catalytic properties, we wondered whether MeHis could allow the development of improved enzymes for more complex chemical transformations. Given the distinctive molecular features of MeHis compared with His (as detailed above), we also envisaged that its use as a catalytic nucleophile could open up new evolutionary pathways during enzyme engineering. To explore these hypotheses, we looked to our recently engineered enzyme (BH32.14) for enantioselective Morita-Baylis-Hillman reactions^13,25^, which involve the coupling of activated alkenes with carbon electrophiles (Fig. 1A). MBH reactions are valuable carbon-carbon bond forming transformations in organic synthesis, for which no natural enzymes are known^26–28^. To develop BH32.14, we subjected a modestly active computational design (BH32) to extensive evolutionary optimization (Fig. 1B), affording a biocatalyst that is orders of magnitude more efficient than analogous small molecule catalysts^13,29^. BH32.14 catalysis relies on a designed His23 nucleophile paired with a flexible Arg124, which emerged during evolution and shuttles between conformational states to stabilize multiple oxyanion intermediates formed along the reaction coordinate.

In this study, we explore the evolutionary trajectory of a BH32 variant with MeHis in place of His23. This engineering not only affords a more efficient MBH enzyme, but interestingly also results in a dramatically altered mechanistic outcome.

## Results

### Evolution of a proficient MBHase with a non-canonical MeHis nucleophile

To identify a suitable starting template for engineering a MeHis-containing MBHase, we replaced the His23 nucleophile of BH32 and selected evolved descendants with MeHis using an engineered pyrrolysyl-tRNA synthetase/tRNA pair^30^. These variants were evaluated for activity towards the MBH coupling of 2-cyclohexen-1-one (**1**) and 4-nitrobenzaldehyde (**2**) (Fig. 1A). In contrast to the improved hydrolytic activity observed upon His23MeHis substitution in BH32^20^, MBH activity was reduced upon MeHis incorporation across all BH32 variants (Fig. 1C, Supplementary Fig. 1). Of the modified variants tested, BH32.8 His23MeHis (subsequently referred to as BH_MeHis_1.0) was found to have the highest activity and was selected for further engineering. It is interesting to note that while evolutionary progression from BH32.8 to BH32.14 led to a 20-fold increase in MBH activity with His as a nucleophile, the analogous progression with MeHis23 led to a reduction in activity (Fig. 1C). BH_MeHis_1.0 was also found to have an altered pH optimum compared to BH32.8, with the highest conversion observed at pH 6.0 (Supplementary Fig. 2).

To improve enzyme activity, BH_MeHis_1.0 was subjected to successive rounds of laboratory evolution. Individual library variants were arrayed in 96-well plates and evaluated as clarified cell lysate using a UPLC assay monitoring conversion of **1** and **2** to MBH adduct **3**. The evolutionary strategy employed a combination of local and global mutagenesis (see Supplementary Table 1). The most active (*ca*. 1%) clones from each round were selected for further evaluation as purified proteins. Beneficial mutations identified in each round were subsequently combined by DNA shuffling.

**Fig. 2:**
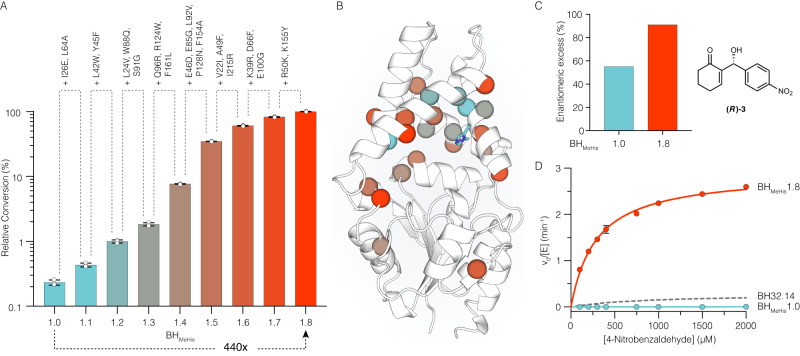
Characterization of BH_MeHis_1.0, BH_MeHis_1.8, and selected variants. **A** Bar chart showing the mean relative conversion achieved along the BH_MeHis_1.8 evolutionary trajectory. Biotransformations were performed using **1** (15 mM), **2** (1.5 mM) and enzyme (1.5 µM) in PBS (pH 6.0) with 3% (v/v) MeCN as cosolvent and analyzed by UPLC following 3 h incubation. Error bars represent the standard deviation of measurements made in triplicate centred around the averaged value. To eliminate errors arising from determination of low conversions, BH_MeHis_1.0 was monitored over a longer timeframe and conversions were interpolated using linear regression. **B** Structure showing the amino acid positions mutated in BH_MeHis_1.8 (PDB: 8BP0, https://www.rcsb.org/structure/8BP0). Mutations represented as spheres at the C_α_ and coloured according to their order of introduction, corresponding to the variants shown in Fig. 2A. MeHis23 is shown as atom-coloured sticks with blue carbon atoms. **C** Bar chart showing the enantiomeric excess of BH_MeHis_1.0 (blue) and BH_MeHis_1.8 (red) towards the (*R*)-enantiomer of MBH product **3**. Reactions performed using BH_MeHis_1.0 (60 µM) or BH_MeHis_1.8 (10 µM) with **1** (15 mM), **2** (1.5 mM), PBS pH 6.0 with 20% (v/v) DMSO as cosolvent and analyzed following 23 h incubation. **D** Michaelis-Menten plot for the MBH reaction between **1** and **2** catalysed by BH_MeHis_1.8 (red), BH_MeHis_1.0 (blue) and BH32.14 (grey dashed)^13^. Assays were performed at a fixed concentration of **1** (25 mM) and varying concentrations of **2** (0.1–2 mM). Data points shown are averages of triplicate measurements with error bars representing standard deviation. Representative Michaelis-Menten plots at fixed concentrations of **1** and **2** are shown in Supplementary Fig. 5. Source data are provided as a Source Data file.

**Fig. 3:**
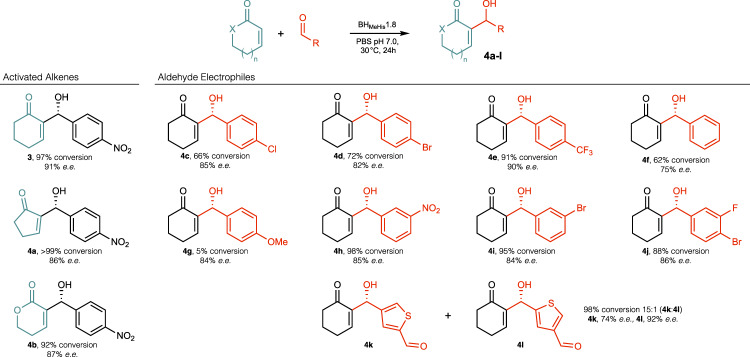
Substrate scope of BH_MeHis_1.8. BH_MeHis_1.8 promotes the MBH reaction with a range of alkene and aldehyde coupling partners with high conversions to product and selectivities. X = CH_2_ or O, n = 0 or 1. Specific reaction conditions are presented in Supplementary Table 6. Source data are provided as a Source Data file.

Following evaluation of >18,000 clones, a BH_MeHis_1.8 variant emerged containing 23 mutations (Fig. 2A, B). The relative activities of variants along the evolutionary trajectory were compared and show how steady improvements in performance have culminated in a variant that is 440-fold more active than BH_MeHis_1.0 (Fig. 2A). This improvement in catalytic performance also correlated with improvements in enantioselectivity, with the (*R*)-enantiomer of **3** formed in 91% *e.e*. with BH_MeHis_1.8 compared with a more modest 55% *e.e*. with the starting variant (Fig. 2C and Supplementary Table 2). Notably, with BH_MeHis_1.8, **3** is formed as the exclusive product with no detectable aldol by-product **S1**, as observed in biotransformations with BH_MeHis_1.0 (Supplementary Fig. 3). Despite performing evolution at pH 6.0, the pH optimum of BH_MeHis_1.8 has increased compared to BH_MeHis_1.0, with maximum conversions achieved at pH 7.0 (Supplementary Fig. 4). More detailed kinetic characterization of BH_MeHis_1.8 reveals a *k*_cat_ of 4.5 ± 0.19 min^−1^, making it 13-fold more active than our previously engineered MBHase BH32.14 (0.35 ± 0.03 min^−1^) and 2000-fold more active than the original BH32 design (0.13 ± 0.01 h^−1^) (Fig. 2D, Supplementary Fig. 5A and Table 3)^13^. To highlight the efficiency of BH_MeHis_1.8, we compared its activity to an analogous small molecule nucleophilic catalyst, *N*-methylimidazole. In assays with **1** (15 mM) and **2** (2 mM) as substrates, BH_MeHis_1.8 achieves 26% conversion within 2 h using only 0.1 mol% enzyme. For comparison, even with 1000-fold higher catalyst loading and a prolonged 24 h reaction time, only 2% conversion to rac-**3** is achieved with *N*-methylimidazole (Supplementary Table 4). BH_MeHis_1.8 can also perform greater than 2500 turnovers, can operate at temperatures up to 55 °C without compromising activity, and readily tolerates 20% DMSO as an organic cosolvent (Supplementary Figs. 6 and 7, Supplementary Table 5). To demonstrate synthetic utility, we performed a preparative-scale biotransformation to produce 500 mg of (*R*)-**3** (96% conversion, 82% isolated yield, 91% *e.e*.) using only 0.1 mol% of BH_MeHis_1.8 (Supplementary Fig. 8 and Supplementary Tables 2 and 4). BH_MeHis_1.8 is also able to promote MBH reactions with a range of alkene and aldehyde coupling partners, to generate a diverse array of MBH products **4a**–**l** (Fig. 3 and Supplementary Table 6). With all substrates tested, BH_MeHis_1.8 shows significantly improved efficiency compared with our previously reported MBHase (BH32.14)^13^. These reactions also generally proceed with good to excellent levels of stereocontrol. Interestingly, with some substrates (**4c**–**j**), prolonged reaction times result in a reduction in product *e.e*. with no notable changes in reaction conversion, consistent with these particular MBH reactions being reversible under the assay conditions. BH_MeHis_1.8 can also perform selective transformations of unsymmetrical dialdehyde substrates with high levels of regio-control (**4k:l**, 15:1), which contrasts with the modest regioselectivity observed with BH32.14 (**4k:l**, 2:1).

### Structure and catalytic mechanism

During evolution of BH_MeHis_1.8, Arg124, which was essential to catalysis in BH32.14^13^, was replaced by a tryptophan. This mutation, identified through random mutagenesis, suggests that a substantially altered catalytic mechanism has emerged. To gain insights into this mechanism, crystal structures of *apo*-BH_MeHis_1.0 and *apo*-BH_MeHis_1.8 were solved (Supplementary Table 7). Efforts to obtain structures complexed with either substrate(s) or product have thus far been unsuccessful. Comparison of the BH_MeHis_1.8 and BH_MeHis_1.0 structures reveals that the 23 mutations installed during evolution cause minimal changes to the overall protein fold (secondary structure root mean square deviation (rmsd) 0.47 Å, Supplementary Fig. 9). These structures also overlay well with our previous crystal structure of BH32.12, which has three mutations compared with BH32.14 (rmsd 1.1 Å). The MeHis nucleophile adopts a single conformation in the structures of BH_MeHis_1.8 and BH_MeHis_1.0, however a notable 120° rotation of the imidazole ring has occurred (Supplementary Fig. 9). In BH_MeHis_1.8, MeHis23 is positioned by an adjacent Trp42 residue installed during evolution. MD simulations of BH_MeHis_1.8 show that Trp42 and MeHis23 are well-ordered (Supplementary Fig. 10), with MeHis well-positioned for catalysis (see QM/MM and MD analysis below). Mutation of Trp42 to Phe results in *ca*. 2.2-fold reduction in activity, consistent with its role in positioning and/or activating the MeHis nucleophile (Supplementary Table 4). These observations are further supported by DFT calculations that predict an electron rich tryptophan can preferentially stabilize charged imidazolium intermediates (Supplementary Fig. 11). Molecular docking of (*R*)-**3** into the *apo*-BH_MeHis_1.8 structure reveals a binding mode with the aromatic nitrobenzene ring sandwiched between Trp124 and Phe132 and the polar 1,3-hydroxyketone motif pointing towards a newly introduced Glu26 (Supplementary Fig. 12). Interestingly, a PROPKA 3^31^ calculation based on *apo*-BH_MeHis_1.8 predicts that Glu26 has an unusually high p*K*_a_ of 8.1, likely due to its positioning within a hydrophobic environment surrounded by non-polar sidechains. For comparison, in BH32.12, the 1,3-hydroxyketone motif of (*R*)-**3** is orientated towards Arg124, with the nitrobenzene ring forming π-stacking interactions with Trp88, which has been mutated to Gln88 in BH_MeHis_1.8^13^.

**Fig. 4:**
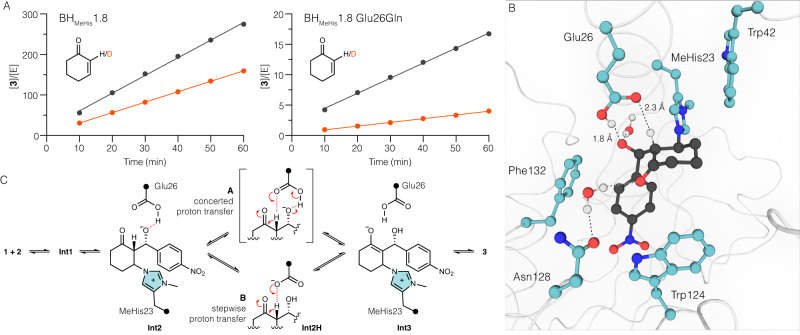
Investigation of the catalytic mechanism of BH_MeHis_1.8. **A** KIE plots showing changes in reaction rate using either 2-cyclohexen-1-one (**1**, black lines) or 2-deuterocyclohex-2-en-1-one (**S2**, red lines) for BH_MeHis_1.8 and BH_MeHis_1.8 Glu26Gln. Reactions were performed using **1** or **S2** (25 mM), **2** (2 mM) in PBS pH 7.0 with 3% (v/v) MeCN as cosolvent. Reactions were performed in triplicate. Source data are provided as a Source Data file. **B** Representative MD snapshot of BH_MeHis_1.8:**Int2** complex with a protonated glutamic acid Glu(H)26 (model **A**) from a 500 ns simulation. **Int2** (black) and key amino acid residues (blue) are shown in ball and stick representation with hydrogen bonds shown as black dashed lines. **C** Proposed mechanisms of BH_MeHis_1.8 showing a concerted (top) and a stepwise (bottom) proton transfer from **Int2** to **Int3** mediated by Glu(H)26.

To explore the role of Glu26 in catalysis, we performed assays with Glu26Gln and Glu26Ala variants of BH_MeHis_1.8. These substitutions led to substantial 20-fold and 100-fold reductions in reaction rates, respectively, underscoring the importance of Glu26 to the catalytic mechanism (Supplementary Fig. 13). While mutation of Glu26 is detrimental for MBH catalysis, it has minimal effect on the rate of reaction with a mechanistic inhibitor designed to report on stabilization of oxyanions at C1 (**Int1** and **Int3**) (Supplementary Fig. 14)^13^. MBH reactions performed with 2-deuterocyclohex-2-en-1-one (**S2**) revealed a kinetic isotope effect (KIE) of 1.7 with BH_MeHis_1.8, which is increased to 4.0 in the Glu26Gln variant (Fig. 4A, Supplementary Table 8). These data suggest that the transition from **Int2** to **Int3** is at least partially rate limiting in both variants and that Glu26 plays an important role in this proton transfer step. Interestingly, inverse solvent KIEs of 0.9 and 0.6 are also observed in BH_MeHis_1.8 and the Glu26Gln variant, respectively (Supplementary Table 8).

To gain further insights into the role of Glu26, we generated two computational models: (**A**). a BH_MeHis_1.8:**Int2** complex with a protonated glutamic acid (Glu(H)26) and (**B**). a BH_MeHis_1.8:**Int2H** complex where the proton has been transferred from Glu(H)26 to **Int2**, and performed MD simulations over 500 ns (Fig. 4B and Supplementary Figs. 15-17). In model A, Glu(H)26 is well-poised to mediate proton transfer from the C2 proton to the C3-alkoxide (Fig. 4B, relevant O-H distance plots are shown in Supplementary Fig. 16C and D). In model B, Glu26 is also well positioned to act as a catalytic base for the rate-limiting C2 deprotonation (Supplementary Figs. 15 and 17C). Taken together, these models further support the importance of Glu26 in promoting proton transfer from **Int2** to generate **Int3**, either through a concerted (model **A**) or stepwise (model **B**) process (Fig. 4C). In the absence of Glu26, we propose that MBH catalysis may proceed through a less effective, water-mediated proton transfer, as previously proposed for small-molecule catalyzed MBH reactions in protic solvents and for our previously engineered BH32.14 enzyme^13,26,28^.

**Fig. 5:**
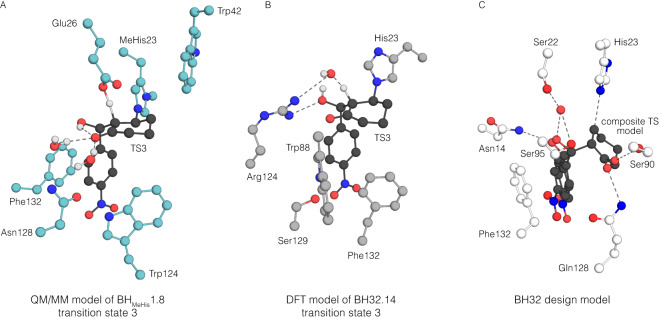
Computational model of BH_MeHis_1.8 and its comparison to existing BH32.14 and BH32 models. **A** A QM/MM model of transition state three in the reaction mechanism of BH_MeHis_1.8 including key active site residues, shown as atom-coloured balls and sticks (ligand shown with black carbon atoms, and active site residues with blue carbon atoms). Glu26 serves as a catalytic base for deprotonation of C2. Trp124 and Phe132 form π-stacking interactions with the electron deficient nitro-arene. Ordered water molecules are shown as balls and sticks. **B** DFT model of transition state 3 in the reaction mechanism of BH32.14^13^ including active site residues shown in atom-coloured balls and sticks (ligand shown with black carbon atoms, and active site residues with grey carbon atoms). Arg124 is shown to stabilize oxyanion intermediates throughout the mechanism. Proton transfer is mediated by an ordered water molecule. **C** Computational design model for BH32^29^. A composite transition state model and surrounding key residues are shown in atom-coloured balls and sticks (ligand shown with black carbon atoms, and active site residues with white carbon atoms). Gln128 was designed to stabilize C1 oxyanion. An ordered water molecule (shown as a red sphere) was designed to stabilize the C3 oxyanion and to mediate proton transfer.

To further analyze proton transfer by Glu(H)26, QM/MM calculations were performed. The p*K*_a_ difference between Glu(H)26 and the C3 alkoxide suggests that model (**B**) is the more likely protonation state for **Int2**, and indeed attempts to optimize a model A structure resulted in proton transfer to generate model (**B**). Deprotonation of the C2 proton of **Int2H** by Glu26 proceeds with a potential energy barrier of 45.1 kJ mol^−1^ (Supplementary Figs. 18 and 19), resulting in an **Int3** state with the C1 oxyanion stabilized by two water molecules and an internal hydrogen bond to the C3-OH (Fig. 5A). This mechanism is in contrast to that of our previously engineered MBHase, BH32.14, where oxyanion intermediates are stabilised by hydrogen bonding to Arg124 and the proton transfer step from **Int2** to **Int3** is mediated by an ordered water molecule (Fig. 5B). The final chemical step involves elimination of the MeHis23 nucleophile to generate MBH product (*R*)-**3**, and has an energy barrier of 46.5 kJ mol^−1^. This step initially generates a product bound state (**P**) 15.4 kJ mol^−1^ above **Int2H**, however repositioning of water and Glu(H)26 creates a significantly lower energy product state (**P**’), that is –44.6 kJ mol^−1^ lower than **Int2H**. Such rearrangement is very facile, and similar rearrangements are observed very quickly (<1 ns) during MD simulations initiated at (**P**) (Supplementary Fig. 20). The calculated energy barriers for the conversion of **Int2H** to **Int3**, and **Int3** to (**P**) are very similar, which is consistent with a partially rate limiting deprotonation step. These calculations are therefore consistent with the experimentally observed KIE of 1.7 being lower than the calculated intrinsic KIE of 4.7 for H/D abstraction. The observed inverse SKIE of 0.9 is also consistent with the computed SKIEs of 0.93 and 0.86 for these two chemical steps, which arises from D_2_O acting as a stronger hydrogen-bond donor in the transition states.

### Significance of the non-canonical MeHis nucleophile

To investigate the importance of the non-canonical nucleophile to BH_MeHis_1.8 catalysis, we generated variants with MeHis replaced by Ala and His. As anticipated, the MeHis23Ala mutation abolishes catalytic activity (Supplementary Table 4). In contrast, introduction of a canonical His23 nucleophile leads to only a 4-fold reduction in activity (*k*_cat_ = 1.13 ± 0.05 min^−1^, Supplementary Fig. 5 and Table 3). Interestingly, this MeHis23His variant is 4-fold more active than our previously engineered MBHase BH32.14 (*k*_cat_ = 0.35 min^−1^)^13^. These data show that in the context of BH_MeHis_1.8, the non-canonical nucleophile is beneficial but not essential to catalytic function. Furthermore, analysis of the BH_MeHis_1.8 evolutionary trajectory reveals that the MeHis nucleophile was important in unlocking the new mechanistic pathway observed in this enzyme (Supplementary Fig. 21). The first four mutations introduced into BH_MeHis_1.0 during rounds 1 and 2 of evolution gave a > 4-fold increase in activity. In contrast, these mutations reduce activity in the analogous His23 containing variant (BH32.8), and therefore would not have been selected if evolving with His23 as the catalytic nucleophile. These mutations include the key catalytic residue Glu26 and Trp42, which plays a role in positioning and/or activating the MeHis23 nucleophile. Interestingly, while the early stages of BH_MeHis_1.8 evolution were strongly linked to the identity of the nucleophile, mutations introduced from round 3 onwards gave activity improvements with either MeHis or His (Supplementary Fig. 21).

## Discussion

In this study, an expanded genetic code has allowed us to explore divergent evolutionary trajectories where the only distinguishing feature was the identity of the key catalytic nucleophile. Interestingly, introduction of a non-canonical nucleophile led to a dramatically altered evolutionary outcome, giving rise to more efficient MBH biocatalysts and providing a new mechanistic solution to the MBH reaction. We note that the evolutionary trajectory taken to BH_MeHis_1.8 would not have been accessible using His23 as the nucleophile. Similarly, the evolutionary pathway leading to our previously engineered MBHase, BH32.14, would not have been taken if MeHis was selected as the nucleophile (Fig. 1C). These observations suggest that replacing key catalytic elements of designed enzymes by subtly altered analogues can open up new evolutionary trajectories that would not have otherwise been explored. Although further work is needed to explore the generalizability of this approach, it is not altogether surprising that the distinctive features of His and MeHis as catalytic nucleophiles could lead to altered selection pressures, and therefore different evolutionary outcomes, during enzyme engineering.

In contrast to our previously designed hydrolase OE1.3, where catalysis was strictly dependent on MeHis^20^, MeHis to His substitution in BH_MeHis_1.8 still gave rise to a potent MBH biocatalyst, albeit with somewhat reduced activity. Given the increased cost of producing enzymes with non-canonical amino acids, this His23 variant could potentially serve as valuable starting point for developing practically useful MBH biocatalysts in the future. Alternatively, the costs associated with producing proteins containing MeHis and other valuable non-canonical amino acids (ncAAs) can be reduced substantially by engineering orthogonal aaRS/tRNA pairs that operate efficiently at low ncAA concentrations, or by developing engineered heterologous hosts that biosynthesize target ncAAs and are specifically optimized for efficient UAG codon suppression^32–34^. In this way we could fully capitalize on the beneficial features of MeHis as a catalytic nucleophile, and more generally on the expanded range of catalytic functions that become accessible with an expanded set of functional amino acid side chains.

It is important to note that the catalytic features of BH_MeHis_1.8 and our previously engineered MBHase BH32.14 are quite distinct from those present in the original BH32 design model (Fig. 5). These discrepancies serve to highlight the complexities of predicting and designing optimal active site arrangements for performing new chemistries in proteins, especially for demanding multi-step transformations such as MBH reactions. Nevertheless, efficient engineered enzymes such as BH_MeHis_1.8 can now serve as the blueprint for the next generation of MBH designs. The challenging, multi-step nature of MBH reactions will undoubtedly provide a rigorous examination of computational design methods. However, given the remarkable accuracy and speed enabled by modern deep-learning based protein design tools, we are optimistic that the design of de novo enzymes that recapitulate the catalytic features of engineered enzymes such as BH_MeHis_1.8 could be within reach^35^.

## Methods

### Materials

All chemicals and biological materials were obtained from commercial suppliers. Lysozyme, DNase I, kanamycin sulphate and chloramphenicol were purchased from Sigma-Aldrich; polymyxin B sulfate from AlfaAesar; LB agar, LB medium, 2 × YT medium and arabinose from Formedium; *Escherichia coli* (*E. coli*) 5α, Q5 DNA polymerase, T4 DNA ligase and restriction enzymes from New England BioLabs; *N*_*δ*_-methylhistidine (MeHis; H-His(3-Me)-OH) from Bachem; *E. coli* DH10B from Thermo Fisher; and oligonucleotides were synthesized by Integrated DNA Technologies.

### Construction of pBbE8k_BH32_His23MeHis and variants

The His23 CAT codon of the BH32 gene and its variants^13^ was mutated to TAG for stop codon suppression using overlap extension PCR (see Supplementary Table 10 for primer sequences). The gene was subcloned using *NdeI* and *XhoI* restriction sites into a pBbE8K vector^36^ containing a C-terminal 6xHis tag to yield pBbE8K_BH32_His23MeHis and its variants. Similarly, MeHis23Ala, MeHis23His, Glu26Ala and Glu26Gln mutations were introducted into BH_MeHis_1.8 to generate pBbE8K_BH_MeHis_1.8_MeHis23Ala, pBbE8K_BH_MeHis_1.8_MeHis23His, pBbE8K_BH_MeHis_1.8_Glu26Ala and pBbE8K_BH_MeHis_1.8_Glu26Gln respectively in the same way. BH_MeHis_1.0, BH_MeHis_1.8 and BH_MeHis_1.8_MeHis23His were also subcloned, using *NdeI* and *XhoI* restriction sites, into a pBbE8K vector modified to include a Strep-tag after the *XhoI* restriction site to yield pBbE8K_BH_MeHis_1.0_Strep, pBbE8K_BH_MeHis_1.8_Strep and pBbE8K_BH_MeHis_1.8_MeHis23His_Strep, respectively.

### Construction of pEVOL_PylRS_MeHis_/tRNA_CUA_

The *Methanosarcina mazei* analogue of *Methanosarcina barkeri* PylRS^30^ (*Mm* Leu305Ile/Tyr306Phe/Leu309Gly/Cys348Phe/Tyr384Phe) was prepared by overlap extension PCR. Two copies of the gene were cloned into pEVOL using *BglII/SalI* and *NdeI/PstI* restriction sites. The vector also contained the *M. mazei* tRNA_CUA_.

### Protein production and purification

For expression of BH32 and its variants and BH_MeHis_1.8 MeHis23Ala/His, chemically competent *E. coli* 5α were transformed with the relevant pBbE8k_BH32 constructs. Single colonies of freshly transformed cells were cultured for 18 h in 5 mL LB medium containing 25 µg mL^−1^ kanamycin sulphate. Starter cultures (500 µL) were used to inoculate 50 mL 2 × YT medium supplemented with 25 µg mL^−1^ kanamycin sulphate. Cultures were grown at 37 °C, 200 r.p.m. to an optical density at 600 nm (OD_600_) of around 0.6. Protein expression was induced with the addition of L-arabinose to a final concentration of 10 mM.

For the expression of BH32_MeHis, BH_MeHis_1.0 and their variants, chemically competent *E. coli* DH10B cells containing pEVOL_PylRS_MeHis_/tRNA_CUA_ were transformed with the appropriate pBbE8K construct. Protein production was carried out as described above with the addition of 25 µg mL^−1^ chloramphenicol and MeHis (10 mM final concentration).

Induced cultures were incubated for 20 h at 25 °C and the cells were subsequently collected by centrifugation (3,220 *g* for 10 min). Pelleted cells were resuspended in lysis buffer (50 mM HEPES, 300 mM NaCl, pH 7.5 containing 20 mM imidazole) and lysed by sonication (10 min, 1 s on/off pulse, 50% intensity). Cell lysates were cleared by centrifugation (27,216 *g* for 30 min), and supernatants were subjected to affinity chromatography using Ni-NTA Agarose (Qiagen). Purified protein was eluted using 50 mM HEPES, 300 mM NaCl, pH 7.5 containing 250 mM imidazole.

For Strep-tagged variants, pelleted cells were resuspended in NP buffer (50 mM NaH_2_PO_4_, 300 mM NaCl, pH 8.0) and lysed by sonication. Cell lysates were cleared by centrifugation (27,216 g for 30 min), supernatants were subjected to a Strep-Tactin Superflow Plus resin (Qiagen), which was washed with 10 column volumes of NP buffer and purified protein was eluted using 50 mM NaH_2_PO_4_, 300 mM NaCl and 2.5 mM desthiobiotin at pH 8.0.

Proteins were desalted using 10DG desalting columns (Bio-Rad) with PBS buffer (pH as stated in reaction conditions) and analysed by SDS-PAGE. Proteins were aliquoted, flash-frozen in liquid nitrogen and stored at −80 °C. Protein concentrations were determined by measuring the absorbance at 280 nm using calculated extinction coefficients (ExPASy ProtParam).

### Mass spectrometry

Purified protein samples were desalted into 0.1% acetic acid using a 10 k MWCO Vivaspin (Sartorius) and diluted to a final concentration of 0.5 mg mL^−1^. Mass spectrometry was performed using a 1200 series Agilent LC, with a 5 µL injection into 5% acetonitrile (with 0.1% formic acid) and desalted inline for 1 min. Protein was eluted over 1 min using 95% acetonitrile with 5% water. The resulting multiply charged spectrum was analysed using an Agilent QTOF 6510 and deconvoluted using Agilent MassHunter Software.

### Library construction

Primer sequences used to generate DNA libraries are shown in Supplementary Table 11.

*Saturation mutagenesis: rounds 1, 3, 5–7, 9-10*. Between 20–28 positions were individually randomised using primers with NNK degenerate codons (Supplementary Table 11). DNA libraries were constructed using overlap extension PCR (templates and targeted positions for each round are summarised in Supplementary Table 1). The linear library fragments were digested using *NdeI* and *XhoI* restriction enzymes and ligated into pBbE8K using T4 DNA ligase.

*Combinatorial active site saturation testing (CASTing): round 2*. A single CASTing library between L42 and Y45 was prepared by overlap extension PCR using pBbE8k_BH_MeHis_1.1 as a template and degenerate primer pairs (22c-trick^37^). The library genes were subcloned as described above.

*Random mutagenesis using error-prone PCR: rounds 4 and 8*. The library was generated by error-prone PCR of the entire gene using an Agilent GeneMorph®II Random Mutagenesis Kit according to the manufacturer’s protocol to generate an average of 2.5 mutations per gene (the templates for rounds 4 and 8 are summarised in Supplementary Table 1). The gene was cloned as described above. Identified ‘hotspots’ were individually randomised in subsequent rounds by saturation mutagenesis (rounds 5 and 9, see above).

### Shuffling by overlap extension PCR

After each round of evolution, beneficial diversity was combined by DNA shuffling of fragments generated by overlap extension PCR. Primers were designed that encoded either the parent amino acid or the identified mutation. These primers were used to generate short fragments (up to 6) which were gel-purified and mixed appropriately in overlap extension PCR to generate genes containing all possible combinations of mutations. Genes were cloned as described above.

### Library screening

For protein expression and screening, all transfer and aliquoting steps were performed using Hamilton liquid-handling robots. Chemically competent *E. coli* DH10B cells containing pEVOL_PylRS_MeHis_/tRNA_CUA_ were transformed with the library plasmids. Freshly transformed clones were used to inoculate 150 μL of 2 x YT medium supplemented with 25 μg mL^−1^ kanamycin sulphate and 25 μg mL^−1^ chloramphenicol in Corning® Costar® 96-well microtiter round bottom plates. For reference, each plate contained 6 freshly transformed clones of the parent template and 2 clones containing an empty pBbE8k vector. Plates were incubated overnight at 30 °C, 80% humidity in a shaking incubator at 850 r.p.m. 20 µL of overnight culture was used to inoculate 480 μL 2 x YT medium supplemented with 25 μg mL^−1^ kanamycin sulphate, 25 μg mL^−1^ chloramphenicol and 10 mM MeHis in 96-deep-well plates. The cultures were incubated at 30 °C, 80% humidity with shaking at 850 r.p.m. until an OD_600_ of about 0.6 was reached, and L-arabinose was added to a final concentration of 10 mM. Induced plates were incubated for 20 h at 30 °C, 80% humidity with shaking at 850 r.p.m. Cells were harvested by centrifugation at 2,900 *g* for 10 min. The supernatant was discarded, and the pelleted cells were resuspended in 400 μL of lysis buffer (PBS pH 6.0, buffer supplemented with 1.0 mg mL^−1^ lysozyme, 0.5 mg mL^−1^ polymixin B and 10 μg mL^−1^ DNase I) and incubated for 2 h at 30 °C, 80% humidity with shaking at 850 r.p.m. Cell debris was removed by centrifugation at 2,900 *g* for 10 min.

*Rounds 1–5*: 75 µL clarified lysate were transferred to 96-well polypropylene microtiter plates. Reactions were initiated with the addition of 25 µL assay mix (2-cyclohexen-1-one **1** (15 mM final concentration), 4-nitrobenzaldehyde **2** (1.5 mM final concentration), 12% (v/v) MeCN in PBS pH 6.0). Assay plates were sealed and incubated for 20 h at 30 °C, 80% humidity with shaking 850 r.p.m. Reactions were quenched with addition of 100 µL MeCN, heat sealed and incubated for 2 h at 850 r.p.m. at 30 °C. Precipitated protein was removed by centrifugation at 2,900 *g* for 10 min. 100 µL of the clarified reactions were transferred to 96-well polypropylene microtiter plates and heat sealed with pierceable foil for UPLC analysis as described below. From round 2 onwards, the amount of lysate and the reaction time were reduced to achieve <10% conversion on average.

*Rounds 6*–*10*: Reaction plates were prepared as above with lower substrate loading. 25 µL of assay mix (2-cyclohexen-1-one **1** (3 mM final concentration), 4-nitrobenzaldehyde **2** (0.6 mM final concentration), 12% (v/v) MeCN in PBS pH 6.0) was added to lysate to initiate the reactions. As above, assay conditions were altered throughout the rounds to keep conversion <10% including reducing reaction time, volume of lysate and increasing lysis volume. For round 10, the lysate was further diluted 2.5-fold. 25 µL of diluted lysate was transferred to a 96-well polypropylene microtiter plate with the addition of 50 µL PBS pH 6.0 and 25 µL assay mix. Reactions were quenched after 2 h and prepared for UPLC analysis as stated above.

Following each round, the top (*ca*. 1%) variants were rescreened in triplicate. Expression and screening were performed as described above but from glycerol stocks prepared from the original overnight culture. Confirmed hits were evaluated in purified protein before shuffling.

### General procedure for analytical scale biotransformations

Analytical scale biotransformations were performed using **1** (15 mM), **2** (1.5–2 mM) and the relevant biocatalyst (1.5–60 µM) in PBS (pH 6.0, 7.0 or 7.4) with 3% (v/v) MeCN (or 20% (v/v) DMSO for *e.e*. measurements) as a cosolvent at 30 °C. For comparison, reactions were also performed with small-molecule catalysts (200 µM, Supplementary Table 4). Following incubation, reactions were quenched with 1 volume MeCN. Quenched reactions were shaken (850 r.p.m) for 2 h. Precipitated protein was removed by centrifugation (14,000 *g* for 10 min) and supernatants were transferred to a fresh plate for UPLC analysis (*see chromatographic analysis*). For SFC analysis, the substrates and products were extracted with 3 volumes of ethyl acetate. Precipitated protein was removed by centrifugation (14,000 *g* for 10 min), the organic phase was separated and directly injected onto the SFC.

### General procedure for substrate scope biotransformations

Biotransformations for substrate scope (Fig. 3) were performed using the specified alkene and aldehyde with BH_MeHis_1.8 (100 µM) in PBS (pH 7.0) with 20% (v/v) DMSO as cosolvent at 30 °C (Supplementary Table 6). Following incubation, reactions were quenched with 1 volume MeCN. Quenched reactions were shaken (850 r.p.m) for 2 h. Precipitated protein was removed by centrifugation (14,000 *g* for 10 min) and supernatant was analysed by UPLC (*see chromatographic analysis*). For SFC analysis, the substrates and products were extracted with 3 volumes of ethyl acetate. Precipitated protein was removed by centrifugation (14,000 *g* for 10 min), the organic phase was separated and directly injected onto the SFC.

### Chromatographic analysis

UPLC analysis was performed on a 1290 Infinity II Agilent LC system with a Kinetex® 5 µm XB-C18 100 Å LC Column, 50 × 2.1 mm (Phenomenex). For library screening an isocratic method using 22% MeCN in water at 1 mL min^−1^ for 2 min was used. Peaks were integrated using Agilent OpenLab software. As 4-nitrobenzaldehyde (**2**) was the limiting reagent, product conversions were calculated using the extinction coefficient of 600 mM^−1^ cm^−1^ for both 4-nitrobenzaldehyde (**2**) and MBH product (**3**). For characterizing the substrate scope, substrates and products (**4a**–**l**) were eluted over 25 min using a gradient of 5-95% acetonitrile in water at 1 mL min^−1^. Peaks were assigned by comparison to chemically synthesized standards and the peak areas were integrated using Agilent OpenLab software. Previously reported extinction coefficients by our lab were used to calculate conversions^13^.

Chiral analysis was performed using an SFC 1290 Infinity II Agilent system. Enantiomers of the MBH product **3** were separated using a Daicel 80S82 CHIRALPAK ® IA-3 SFC column, 3 mm, 50 mm, 3 µm, and an isocratic method with 35% methanol in CO_2_ at 1 mL min^−1^ for 1 min. For characterizing the substrate scope, previously reported methods were used^13^. Peaks were integrated using Agilent OpenLabs software for calculation of enantioselectivity.

### Kinetic characterization

Initial velocity (*v*_0_) *vs* [4-nitrobenzaldehyde] kinetic data were measured using Strep-tagged purified enzyme (60 µM BH_MeHis_1.0, 0.5 µM BH_MeHis_1.8 MeHis23His and 0.25 µM BH_MeHis_1.8), a fixed concentration of **1** (25 mM) and varying concentrations of **2** (0.1–2 mM). Reactions were performed using PBS pH 7.0 with 3% (v/v) MeCN and were incubated at 30 °C with shaking (850 r.p.m.). BH_MeHis_1.8 and BH_MeHis_1.8 MeHis23His were sampled at 10-min intervals for 1 h and after 75 and 90 min. BH_MeHis_1.0 was sampled every hour from 2 h to 7 h. Samples were quenched with MeCN and analyzed by UPLC as described above (see *chromatographic analysis*).

*v*_0_ vs [2-cyclohexen-1-one] kinetic data were measured using a fixed concentration of **2** (2 mM) and varying concentrations of **1** (2-25 mM) as described above.

Linear fits of conversion *vs* time allowed determination of *v*_0_ (Supplementary Fig. 5). The combined *v*_0_ *vs* [4-nitrobenzaldehyde] and *v*_0_ vs [2-cyclohexen-1-one] steady state kinetic data were fitted globally using the random order binding model (Eq. 1)

1 $$v={k}_{{{{{{\rm{cat}}}}}}}[{{{{{\rm{E}}}}}}][{{{{{\rm{A}}}}}}][B]/(({K}_{{{{{{\rm{mA}}}}}}}+[{{{{{\rm{A}}}}}}])({K}_{{{{{{\rm{mB}}}}}}}+[{{{{{\rm{B}}}}}}]))$$

Where *k*_cat_ corresponds to the turnover number, [E] is the total enzyme concentration, [A] and [B] are the initial 2-cyclohexen-1-one and 4-nitrobenzaldehyde concentrations respectively, *K*_mA_ and *K*_mB_ are the corresponding *apparent* Michaelis constants. Kinetic constants are shown in Supplementary Table 3.

### Total turnover numbers

Total turnover numbers achieved by BH_MeHis_1.8 were determined as follows. BH_MeHis_1.8 (0.1, 0.05 or 0.01 mol%) catalyzed biotransformations were performed in glass vials using **1** (50 mM) and **2** (10 mM) in PBS (pH 7.0) with 20% (v/v) DMSO cosolvent (Supplementary Fig. 6). Reactions were incubated at 30 °C with shaking (850 r.p.m.) and samples were taken at 4, 8.5, 24, 32.5 and 72 h. For UPLC analysis, reactions were quenched at the stated time points with the addition of 1 volume MeCN. Samples were vortexed and precipitated proteins were removed by centrifugation (14,000 *g* for 10 min) followed by UPLC analysis.

### Cosolvent tolerance

To investigate cosolvent tolerance, analytical scale biotransformations were performed using **1** (15 mM), **2** (1.5 mM) and BH_MeHis_1.8 (3 µM) in PBS pH 7.0 with either 3%, 5%, 10%, 15%, 20%, 30%, 40% and 50% (v/v) MeCN or DMSO as cosolvent (Supplementary Table 5). All reactions were incubated at 30 °C and shaken (850 r.p.m.) for 2 h. Reactions were quenched with 1 volume MeCN, shaken (850 r.p.m.) for 2 h, centrifuged (14,000 *g* for 10 min) and analyzed by UPLC.

### Temperature profile

To evaluate the activity of BH_MeHis_1.8 at elevated temperatures (Supplementary Fig. 7) analytical scale biotransformations were performed using **1** (15 mM), **2** (1.5 mM) and BH_MeHis_1.8 (3 µM) in PBS pH 7.0 with 3% (v/v) MeCN as a cosolvent. Enzyme solutions were pre-incubated at the required temperature (25–80 °C at 5 °C intervals) for 15 min prior to initiation by substrate addition. Reactions were quenched with 1 volume MeCN, shaken (850 r.p.m.) for 2 h, centrifuged (14,000 *g* for 10 min) and analyzed by UPLC.

### pH profile

To determine the pH optimum for BH32.8, BH_MeHis_1.0, BH_MeHis_1.8 and BH32.14, (Supplementary Figs. 2 and 4) analytical scale biotransformations were performed using **1** (15 mM), **2** (1.5 mM) and enzyme (3 µM BH_MeHis_1.8, 30 µM BH32.14 or 60 µM BH32.8 and BH_MeHis_1.0) with 3% (v/v) MeCN as cosolvent over a range of pH values (pH 5.8-pH 8.0) in PBS. All reactions were incubated at 30 °C and shaken (850 r.p.m.) for either 2 h (for BH_MeHis_1.8 and BH32.14) or 21 h (for BH32.8 and BH_MeHis_1.0). Reactions were quenched with 1 volume MeCN, shaken (850 r.p.m.) for 2 h, centrifuged (14,000 *g* for 10 min) and analyzed by UPLC.

### Kinetic Isotope Effects (KIE) and Solvent Kinetic Isotope Effects (SKIE)

KIE and SKIE experiments were performed in PBS pH/pD 7.0 (Fig. 4A, Supplementary Table 8). Deuterated buffers were prepared using 99.9% D_2_O with pD adjusted according to the following relationship: pD = pH_obs_ + 0.38. Analytical scale biotransformations were performed using **1** or **S2** (25 mM), **2** (2 mM) and the relevant biocatalyst (1 µM BH_MeHis_1.8, 3 µM BH_MeHis_1.8 MeHis23His, 10 µM BH_MeHis_1.8 Glu26Gln) in both deuterated and non-deuterated PBS buffer with 3% (v/v) MeCN as cosolvent. Reactions were performed in triplicate. Reactions in deuterated buffer contained <1% H_2_O final concentration. All reactions were incubated at 30 °C with shaking (850 r.p.m.) with samples taken every 10 min for 1 h. For UPLC analysis, reactions were quenched by the addition of 1 volume of MeCN, shaken (850 r.p.m.) for 2 h and centrifuged (14,000 *g*) for 10 min.

### Inhibition assay

Stopped-flow absorbance experiments were performed on an Applied Photophysics SX18 stopped-flow spectrophotometer (Applied Photophysics Ltd., Leatherhead, UK) equipped with a xenon arc lamp and a 1 cm path length in PBS, pH 7.0 buffer. To follow inhibitor binding, a single mixing experiment was performed whereby the drive syringes were loaded with the respective enzyme variant (10 µM) and inhibitor (25 µM). Data was collected at 325 nm at RT using a (PDA) detector and XSCAN software.

### Preparative-scale biotransformation

A preparative-scale biotransformation was performed using **1** (50 mM), **2** (10 mM), Strep-tag purified BH_MeHis_1.8 (10 µM) in PBS (pH 7.0, 200 mL) with 20% DMSO (50 mL) as a cosolvent. The reaction was incubated at 30 °C with shaking at 100 r.p.m. for 13 h. An aliquot (100 µL) was removed and quenched with MeCN for UPLC analysis, which showed the reaction had proceeded to 96% conversion. The reaction mixture was extracted with ethyl acetate (2 × 400 mL), dried over MgSO_4_, filtered and the solvent was removed *in vacuo*. The crude product (Supplementary Fig. 8) was purified by flash column chromatography (5:1 cyclohexane:ethyl acetate) to give 2-(hydroxy(4-nitrophenyl)methyl)cyclohex-2-en-1-one, **3** as a light yellow solid (505 mg, 82%). Spectral data is consistent with literature values^38^. δ_H_ (400 MHz, CDCl_3_): 8.20 (m, 2H), 7.55 (m, 2H), 6.80 (t, *J* = 4.1 Hz, 1H), 5.61 (s, 1 H), 3.44 (br s, 1H), 2.45 (m, 4H), 2.02 (m, 2H).

### Preparation of product standards 3, S1 and 4a-l

All product standards were prepared using the same general procedure as previously reported^13^. Preparation of MBH product **3** afforded aldol side product **S1**.

#### 2-(hydroxy(4-nitrophenyl)methyl)cyclohex-2-en-1-one (3)

(562 mg, 23%). Spectral data is consistent with literature values^39^. δ_H_ (400 MHz, CDCl_3_): 8.20 (m, 2H), 7.55 (m, 2H), 6.80 (t, *J* = 4.1 Hz, 1H), 5.61 (s, 1 H), 3.44 (br s, 1H), 2.45 (m, 4H), 2.02 (m, 2H). ESI+ *m*/*z* = 270 ([M +Na]^+^,100).

#### 6-(hydroxy(4-nitrophenyl)methyl)cyclohex-2-en-1-one (S1)

(180 mg, 7%) as a 4:1 mixture of diastereoisomers. Spectral data is consistent with literature values^39^. δ_H_ (400 MHz, CDCl_3_): 8.26–8.20 (m, 2H), 7.57–7.51 (m, 2H), 7.10–6.97 (m, 1H), 6.13–6.08 (m, 1H), 5.70 (d, *J* = 2.3 Hz, 1H_maj_), 4.99 (d, *J* = 8.7 Hz, 1H_min_), 4.95 (br s, OH_min_), 2.95 (br s, OH_maj_), 2.72–2.65 (m, 1H_maj_), 2.62–2.53 (m, 1H_min_), 2.48–2.25 (m, 2H), 2.06−1.93 (m, 1H), 1.57−1.46 (m, 1H). ESI+ m/z = 270 ([M +Na]^+^,100).

#### 2-((4-nitrophenyl)(hydroxy)methyl)cyclopent-2-en-1-one (4a)

(62 mg, 8%). The spectral data are consistent with literature values^40^. ^1^H NMR (400 MHz, CDCl_3_) δ 8.25–8.20 (m, 2H), 7.62–7.57 (m, 2H), 7.29 (td, *J* = 2.8, 1.2 Hz, 1H), 5.68 (s, 1H), 3.56 (s, 1H), 2.67–2.61 (m, 2H), 2.52–2.46 (m, 2H). ^13^C NMR (101 MHz, CDCl_3_) δ 209.5, 159.9, 148.6, 147.9, 146.8, 127.2, 123.9, 69.3, 35.3, 26.9. ESI+ *m*/*z* = 216 ([M − OH]^+^, 100).

#### 3-(hydroxy(4-nitrophenyl)methyl)−5,6-dihydro-2H-pyran-2-one (4b)

(23 mg, 9%). The spectral data are consistent with literature values^41^. ^1^H NMR (400 MHz, CDCl_3_) δ 8.27–8.15 (m, 2H), 7.64–7.54 (m, 2H), 6.77 (t, *J* = 4.3 Hz, 1H), 5.66 (d, *J* = 4.9 Hz, 1H), 4.44–4.33 (m, 2H), 3.63 (d, *J* = 5.5 Hz, 1H), 2.62–2.47 (m, 2H). ^13^C NMR (101 MHz, CDCl_3_) δ 164.5, 148.3, 147.6, 141.8, 134.2, 127.5, 123.8, 71.8, 66.5, 24.3. ESI− *m*/*z* = 248 ([M − H]^−^, 100).

#### 2-((4-chlorophenyl)(hydroxy)methyl)cyclohex-2-en-1-one (4c)

(114 mg, 15%). The spectral data are consistent with literature values^40^. ^1^H NMR (400 MHz, CDCl_3_) δ 7.30–7.22 (m, 4H), 6.74 (t, *J* = 4.3 Hz, 1H), 5.48 (s, 1H), 3.46 (br s, 1H), 2.45–2.32 (m, 4H), 1.95 (apparent quintet (app quint), *J* = 6.3 Hz, 2H). ^13^C NMR (101 MHz, CDCl_3_) δ 200.4, 147.6, 140.8, 140.4, 133.2, 128.5, 127.9, 71.8, 38.5, 25.8, 22.5. ESI+ *m*/*z* = 221.0564 ([M ^37^Cl − OH]^+^, 30), 219 ([M ^35^Cl − OH]^+^, 100).

#### 2-((4-bromophenyl)(hydroxy)methyl)cyclohex-2-en-1-one (4d)

(128 mg, 14%). The spectral data are consistent with literature values^40^. ^1^H NMR (400 MHz, CDCl_3_) δ 7.49–7.44 (m, 2H), 7.25–7.21 (m, 2H), 6.73 (t, *J* = 4.2 Hz, 1H), 5.50 (s, 1H), 2.56–2.28 (m, 4H), 2.09–1.89 (m, 2H). ^13^C NMR (101 MHz, CDCl_3_) δ 200.5, 147.7, 140.9, 140.8, 131.6, 128.3, 121.5, 72.3, 38.7, 25.9, 22.6. ESI+ *m*/*z* = 265.0070 ([M ^81^Br − OH]^+^, 100), 263 ([M ^79^Br − OH]^+^, 91).

#### 2-((4-(trifluoromethyl)phenyl)(hydroxy)methyl)cyclohex-2-en-1-one (4e)

(196 mg, 22%). The spectral data are consistent with literature values^42^. ^1^H NMR (400 MHz, CDCl_3_) δ 7.58 (d, *J* = 8.1 Hz, 2H), 7.47 (d, *J* = 8.2 Hz, 2H), 6.77 (t, *J* = 4.3 Hz, 1H), 5.57 (d, *J* = 5.4 Hz, 1H), 3.59 (d, *J* = 5.7 Hz, 1H), 2.48–2.37 (m, 4H), 2.04–1.95 (m, 2H). ^13^C NMR (101 MHz, CDCl_3_) δ 200.4, 147.9, 145.9, 140.7, 129.6 (q, *J* = 32.3 Hz), 126.8, 125.4 (q, *J* = 3.8 Hz), 122.8, 72.4, 38.6, 25.9, 22.6. ESI+ *m*/*z* = 253 ([M − OH]^+^, 100).

#### 2-(hydroxy(phenyl)methyl)cyclohex-2-en-1-one (4 f)

(232 mg, 9%). The spectral data are consistent with literature values^39^. ^1^H NMR (400 MHz, CDCl_3_) δ 7.38 – 7.31 (m, 5H), 6.73 (t, J = 4.2 Hz, 1H), 5.56 (s, 1H), 2.52 – 2.41 (m, 2H), 2.41 – 2.34 (m, 2H), 2.09–1.90 (m, 2H).^13^C NMR (101 MHz, CDCl_3_) δ 200.65, 147.59, 141.72, 141.16, 128.46, 127.64, 126.60, 72.75, 38.72, 25.90, 22.65.

#### 2-(hydroxy(4-methoxyphenyl)methyl)cyclohex-2-en-1-one (4 g)

(440 mg, 19%). The spectral data are consistent with literature values^43^. ^1^H NMR (400 MHz, CDCl_3_) δ 7.30–7.25 (m, 2H), 6.90–6.85 (m, 2H), 6.74 (t, *J* = 4.2 Hz, 1H), 5.51 (s, 1H), 3.80 (s, 3H), 3.35 (br s, 1H), 2.48–2.42 (m, 2H), 2.42–2.35 (m, 2H), 2.03–1.96 (m, 2H). ^13^C NMR (100 MHz, CDCl_3_) δ 200.4, 158.9, 147.0, 141.1, 133.8, 127.7, 113.7, 72.0, 55.2, 38.5, 25.7, 22.5. ESI+ *m*/*z* = 255 ([M +Na]^+^,100).

#### 2-((3-nitrophenyl)(hydroxy)methyl)cyclohex-2-en-1-one (4 h)

(132 mg, 16%). The spectral data are consistent with literature values^40^. ^1^H NMR (400 MHz, CDCl_3_) δ 8.21–8.18 (m, 1H), 8.09 (dd, *J* = 8.3, 1.2 Hz, 1H), 7.70 (d, *J* = 7.6 Hz, 1H), 7.49 (t, *J* = 7.9 Hz, 1H), 6.86 (t, *J* = 4.2 Hz, 1H), 5.58 (d, *J* = 5.7 Hz, 1H), 3.67 (d, *J* = 5.8 Hz, 1H), 2.48–2.37 (m, 4H), 2.00 (app quint, *J* = 6.3 Hz, 2H). ^13^C NMR (101 MHz, CDCl_3_) δ 200.2, 148.4, 148.2, 144.4, 140.3, 132.7, 129.3, 122.5, 121.4, 71.9, 38.5, 25.9, 22.5. ESI+ *m*/*z* = 230 ([M − OH]^+^, 100).

#### 2-((3-bromophenyl)(hydroxy)methyl)cyclohex-2-en-1-one (4i)

(67 mg, 7%). The spectral data are consistent with literature values^40^. ^1^H NMR (400 MHz, CDCl_3_) δ 7.55–7.47 (m, 1H), 7.42–7.36 (m, 1H), 7.31–7.26 (m, 1H), 7.20 (t, *J* = 7.8 Hz, 1H), 6.76 (t, *J* = 4.2 Hz, 1H), 5.50 (d, *J* = 5.3 Hz, 1H), 3.48 (d, *J* = 5.6 Hz, 1H), 2.48–2.38 (m, 4H), 2.04–1.96 (m, 2H). ^13^C NMR (101 MHz, CDCl_3_) δ 200.4, 147.9, 144.3, 140.7, 130.7, 130.0, 129.6, 125.2, 122.7, 72.2, 38.6, 25.9, 22.6. ESI+ *m*/*z* = 265 ([M ^81^Br − OH]^+^, 100), 263 ([M ^79^Br − OH]^+^, 94).

#### 2-((3-fluoro-4-bromophenyl)(hydroxy)methyl)cyclohex-2-en-1-one (4j)

(43 mg, 4%). ^1^H NMR (400 MHz, CDCl_3_) δ 7.48 (dd, *J* = 8.3, 7.0 Hz, 1H), 7.16–7.11 (m, 1H), 7.01 (dd, *J* = 8.2, 2.0 Hz, 1H), 6.79 (t, *J* = 4.1, 1H), 5.46 (s, 1H), 3.50 (br s, 1H), 2.49–2.35 (m, 4H), 2.06–1.92 (m, 2H). ^13^C NMR (101 MHz, CDCl_3_) δ 200.3, 159.1 (d, *J* = 247.4 Hz), 147.9, 144.1 (d, *J* = 6.2 Hz), 140.4, 133.3, 123.3 (d, *J* = 3.3 Hz), 114.7 (d, *J* = 23.1 Hz), 107.8 (d, *J* = 20.9 Hz), 71.7 (d, *J* = 1.6 Hz), 38.6, 25.9, 22.5. ESI+ *m*/*z* = 283 ([M^81^Br −OH]^+^, 95), 281 ([M^79^Br −OH]^+^, 100).

#### 4-(hydroxy(6-oxocyclohex-1-en-1-yl)methyl)thiophene-2-carbaldehyde (4k) and 5-(hydroxy(6-oxocyclohex-1-en−1-yl)methyl)thiophene-3-carbaldehyde (4 l) 4k

was obtained as a yellow oil (10 mg, 2%). ^1^H NMR (400 MHz, CDCl_3_) δ 9.88 (d, *J* = 1.2 Hz, 1H), 7.69 (d, *J* = 1.5 Hz, 1H), 7.64 (m, 1H), 6.86 (t, *J* = 4.2 Hz, 1H), 5.57 (s, 1H), 2.54–2.39 (m, 4H), 2.09–1.96 (m, 2H). ^13^C NMR (101 MHz, CDCl_3_) δ 200.49, 183.12, 147.70, 145.20, 144.2, 140.23, 135.10, 131.11, 69.70, 38.65, 25.89, 22.59. ESI+ *m*/*z* = 219([M − OH]^+^, 100). **4** **l** was obtained as a yellow oil (23 mg, 3%). ^1^H NMR (400 MHz, CDCl_3_) δ 9.81 (s, 1H), 8.02 (d, *J* = 1.3 Hz, 1H), 7.28–7.24 (m, 1H), 6.96 (t, *J* = 4.0 Hz, 1H), 5.63 (br s, 1H), 2.54–2.38 (m, 4H), 2.08–1.99 (m, 2H). ^13^C NMR (101 MHz, CDCl_3_) δ 200.5, 185.3, 149.7, 148.4, 142.8, 139.4, 136.9, 121.4, 70.2, 38.6, 25.9, 22.5. ESI+ *m*/*z* = 219 ([M − OH]^+^, 100), 191 ([M − OH − CO]^+^, 15).

### Preparation of chiral standards

The enantiomers of **3** were separated by preparative chiral HPLC by Reach Separations (Nottingham) to afford (*R*)-**3** (99.5% e.e.) and (*S*)-**3** (99.9% e.e.) as white solids. The absolute stereochemistry was determined by measuring the optical rotation ((*R*)-**3** ( − 52.5°) and (*S*)-**3** ( + 50.0°) at 0.008 g ml^−1^ in dichloromethane (DCM) at 27 °C) and comparison to literature values^44^.

### Preparation of 2-Deutero-cyclohex-2-en−1-one (S2)

2-Deutero-cyclohex-2-en−1-one (**S2**) was prepared in a 4-step synthesis via intermediates **S3-5** detailed below.

*Preparation of 2-Bromo-cyclohex-2-en−1-one (****S3****)*: To a stirred solution of 2-cyclohexen−1-one (3.0 mL, 31.0 mmol) in dichloromethane (80 mL) at 0°C, a mixture of bromine (1.42 mL, 13.6 mmol) in dichloromethane (80 mL) was added dropwise over 1.5 h. Triethylamine (7.2 mL, 51.8 mmol) was added in a single portion and the reaction was warmed to room temperature and stirred for 1.5 h. The reaction mixture was quenched with 1 M HCl (50 mL), the organic phase was washed with brine (50 mL), dried over MgSO_4_, filtered and solvent was removed *in vacuo*. **S3** was afforded as a brown crystalline solid (5.40 g, quant.) that was used in the subsequent step without purification. Spectral data is consistent with literature values^45^. δ_H_ (400 MHz, CDCl_3_): 7.39 (t, *J* = 1.5 Hz, 1H), 2.65-2.52 (m, 2H), 2.49-2.35 (m, 2H), 2.12−1.95 (m, 2H). ^13^C NMR (101 MHz, CDCl_3_): δ 191.3, 151.4, 123.8, 38.4, 28.4, 22.7.

*Preparation of 6-Bromo-1,4-dioxaspiro[4.5]dec-6-ene (****S4****)*: A mixture of 2-bromocyclohex-2-en-1-one (5.40 g, 30.8 mmol), toluene (154 mL), *p-*toluenesulfonic acid (290 mg, 1.54 mmol) and ethylene glycol (3.43 mL, 61.6 mmol) was heated to reflux under Dean-Stark setup for 2.5 h. The reaction was cooled, extracted with NaHCO_3_ (75 mL), washed with brine, dried over MgSO_4_, filtered and concentrated *in vacuo*. The crude product was purified by flash chromatography (0:100 – 6:94 Et_2_O:hexane) to afford **S4** as a colourless oil (3.34 g, 49%). Spectral data is consistent with literature values^46^. δ_H_ (400 MHz, CDCl_3_): 6.38-6.31 (m, 1H), 4.26–4.14 (m, 2H), 4.05–3.93 (m, 2H), 2.14–2.06 (m, 2H), 1.97−1.89 (m, 2H), 1.88–1.74 (m, 2H). ^13^C NMR (101 MHz, CDCl_3_): 136.3, 124.8, 106.0, 66.0, 35.8, 27.7, 20.5.

*Preparation of 6-Deutero-1,4-dioxaspiro[4.5]dec-6-ene (****S5****)*: A solution of 6-bromo-1,4-dioxaspiro[4.5]dec-6-ene (2.44 g, 11.1 mmol) in dry THF (111 mL) was cooled to -78°C under N_2_. *n-*Butyl lithium (6.2 mL, 15.5 mmol) was added dropwise and the resultant mixture was stirred at -78 °C for 1 h before addition of MeOD-*d*_4_ (4.5 mL, 0.111 mol, 99.8% D). The reaction was warmed to room temperature over 1 h, quenched with saturated NH_4_Cl (10 mL) and extracted with Et_2_O (50 mL x 3). Organic fractions were combined, washed with brine, dried over MgSO_4_, filtered and concentrated *in vacuo*. The crude product (1.51 g, 97%) was used directly in the next step without further purification. Key product peaks in the ^1^H NMR spectrum matched the literature data^46^. δ_H_ (400 MHz, CDCl_3_): 5.96 (s, 1H), 4.03–3.91 (m, 4H), 2.13–1.95 (m, 2H), 1.92–1.68 (m, 4H). ^13^C NMR (101 MHz, CDCl_3_): 132.95, 105.8, 64.6, 33.7, 25.0, 20.9.

*Preparation of 2-Deutero-cyclohex-2-en-1-one (****S2****)*: A mixture of oxalic acid (2.6 g, 28.2 mmol), H_2_O (60 mL), 6-deutero-1,4-dioxaspiro[4.5]dec-6-ene (1.27 g, 9.01 mmol) and dichloromethane (60 mL) was vigorously stirred for 3 h at room temperature. The organic phase was removed and the aqueous phase extracted with Et_2_O (75 mL x 3). The organic layers were combined, washed with NaHCO_3_ (30 mL) and brine, dried over MgSO_4_ then filtered and dried *in vacuo*. Purification via flash chromatography (3:97 Et_2_O:dichloromethane) afforded **S2** as a pale yellow oil (720 mg, 82% w/ 20% w/w dichloromethane, 93% D-incorporation). Spectral data consistent with literature values^47^. δ_H_ (400 MHz, CDCl_3_): 7.02-6.92 (m, 1H), 6.00 (dt, *J* = 10.1, 2.1 Hz, 0.07 H, non-D product), 2.45–2.37 (m, 2H), 2.37–2.29 (m, 2H), 2.06–1.95 (m, 2H). ^13^C NMR (101 MHz, CDCl_3_): 199.9, 150.8, 150.7, 130.0 (non-D product), 129.7 (t, *J* = 25.3 Hz), 129.5, 38.2, 27.0, 25.8 (non-D product), 25.7, 22.8.

### Crystallization, refinement and model building

Crystals of BH_MeHis_1.0 and BH_MeHis_1.8 were prepared by mixing 200 nl of 7 mg ml^−1^ protein in 50 mM HEPES pH 7.0 with an equal volume of precipitant. Crystallization conditions were identified using the SG1 screen (Molecular Dimensions). Crystallization conditions for BH_MeHis_1.0: 0.1 M sodium citrate, 0.1 M magnesium acetate tetrahydrate, 29% (w/v) PEG 4000, pH 6.5. Crystallization conditions for BH_MeHis_1.8: 0.2 M magnesium chloride hexahydrate, 0.1 M Bis-Tris, pH = 6.5, 25% PEG 3350. All trials were conducted by sitting-drop vapour diffusion and incubated at 4 °C. Prior to data collection crystals were cryo-protected by the addition of 20% PEG 400 to the mother liquor and plunge cooled in liquid nitrogen. All data were collected at Diamond Light Source (Harwell, UK) using beamline i03. Data reduction was performed with Dials and the structure solved by molecular replacement using a search model derived from PDB: 7O1D. Iterative rounds of model building and refinement were performed in COOT and Phenix.refine, respectively^48^. Validation with MOLPROBITY^49^ and PDBREDO^50^ were incorporated into the iterative rebuild and refinement process. Data collection and refinement statistics are shown in Supplementary Information Table 7. The coordinates and structure factors have been deposited in the Protein Data Bank under accession numbers 8BP1 (https://www.rcsb.org/structure/8BP1) and 8BP0 for BH_MeHis_1.0 and BH_MeHis_1.8, respectively.

### Molecular docking

Molecular docking was performed using MolsoftICM64-Pro (version 3.9–2d). The protein was kept rigid during docking. For docking of product (*R*)-**3** into BH_MeHis_1.8 a distance restraint of 4 Å between the MeHis23 and the position of nucleophilic attack was imposed on the calculation to ensure a productive pose for catalysis (weighting 3).

### Molecular dynamics simulations

A model of BH_MeHis_1.8:**Int2** complex with a protonated glutamic acid (Glu(H)26) (model A) was initially built based on the docked product state. After MD simulation of model A, the ideal position for the protonation of the **Int2** C3 alkoxide (Fig. 4) prompted the simulation of BH_MeHis_1.8:**Int2H** complex where the proton has been transferred from Glu(H)26 to **Int2** (model B). Model B was created by modifying the structure model A after 100 ns of MD. Models of apo BH_MeHis_1.8 and BH_MeHis_1.8 product **P** complexes were also built from the crystal structure (PDB: 8BP0, https://www.rcsb.org/structure/8BP0) and the energy-minimised QM/MM model of the **P** state, respectively. The protonation state of titratable residues was calculated using PROPKA3^31^, and bonding parameters for the MeHis23_**Int2** adducts and product were generated using the AmberTools ANTECHAMBER^51^ module with charges parameterized by RESP fitting to the HF/6-31 G(d,p) electron density of a B3LYP/6-31 + G(d,p) structure optimized in Gaussian16 Revision C.01^52^. MD simulations of model A were then carried out using Gromacs 2018^53,54^ with the Amber14 force field^55^ with a solvation box with a minimum 10 Å buffering distance around the protein and counter-ions generated using AmberTools, retaining crystallographic waters, for a total of 59,405 atoms. Simulations were performed using constant temperature (velocity-rescaling thermostat^56^, 300 K) and pressure (Parrinello-Rahman barostat^57^, 1 bar), 10 Å van der Waals and electrostatic cut-offs, particle mesh Ewald for long-range electrostatics, LINCS bond constraints^58^, periodic boundary conditions and a 2 fs timestep. The protocol for running simulations was as follows: (i) energy minimisation with (a) 10 kJ mol^−1^ Å^−2^ constraints on the protein, (b) 1 kJ mol^−1^ Å^−2^ constraints on the protein, (c) 1 kJ mol^−1^ Å^−2^ constraints on the backbone, (d) no constraints; (ii) 200 ps constant volume (NVT) equilibration of the solvent with 10 kJ mol^−1^ Å^−2^ constraints on the protein; (iii) four 200 ps constant pressure (NPT) equilibration stages with the same decreasing position constraints as for the minimizations; (iv) 500 ns of unconstrained production MD (250 ns of unconstrained production MD for the apo BH_MeHis_1.8 model and 3 × 50 ns of unconstrained production MD for the BH_MeHis_1.8 product **P** model). RMSD calculations were performed using heavy atoms in the protein backbone and side chains.

### Modelling Trp42 stabilisation of imidazole vs imidazolium

In order to estimate the stabilizing effect of W42 on imidazolium formation, we compared the energies of a simple methyl histidine and methyl histidine-cyclohexanone adduct (**Int1**) models with and without adjacent tryptophan analogue (methyl indole), energy minimized either constrained to the geometry from the QM/MM **Int3** structure, or without constraints (Supplementary Fig. 11). The stabilization of the imidazolium over imidazole (**Int1** over MeHis) is ΔΔ*E* = Δ*E*_*2*_ *-* Δ*E*_*1*_, with a negative value indicating preferential stabilization of the imidazolium:

2 $$\Delta {E}_{1}=E({{{{{\rm{Int}}}}}}1)-E({{{{{\rm{MeHis}}}}}})$$

3 $$\Delta {E}_{2}=E({{{{{\rm{Int}}}}}}1{{{{{\rm{\cdot W}}}}}})-E({{{{{\rm{MeHis\cdot W}}}}}})$$

Since the tryptophan stabilization will be governed by π-effects, we performed DFT calculations with the ωB97XD functional^59,60^ as well as MP2^61^ calculations. ωB97XD is a parametrised functional which uses implicit dispersion corrections and performs well for noncovalent interactions including π–π interactions, and MP2 implicitly takes dispersion into account unlike DFT methods. The 6-311 + + G(d,p) basis sets were used for all atoms, and basis set superposition error was calculated using the Counterpoise method^62^, and an implicit water solvation model was used.

From these calculations ΔΔ*E* = −5.0 and −5.1 kJ mol^−1^ (ωB97XD and MP2, respectively) for the constrained models and ΔΔ*E* = −12.2 and −8.3 kJ mol^−1^ for the unconstrained models. The difference in geometry between the constrained and unconstrained models is not significant (RMSD = 1.01 and 1.05 Å for ωB97XD and MP2, respectively for the methyl indole-imidazolium models), and is similar to changes that can be expected during dynamics and catalysis.

We also created unconstrained W42F models, by replacing the methyl indole with toluene, which resulted in ΔΔ*E* = −8.7 and −5.7 kJ mol^−1^ (ωB97XD and MP2, respectively).

### QM/MM calculations

The system was first prepared by removing water molecules >25 Å from Glu26 or the MeHis23 adduct of **Int2H** and all counter-ions, for a total system size of 12,451 atoms. The QM region was then defined as Glu26, the MeHis23 adduct of **Int2H** and the 6 nearest water molecules (totalling 75 atoms), and link atoms were placed between the Cα and Cβ atoms of MeHis23 and Glu26. All residues with at least one atom within an 18 Å radius of the **Int2H** C1 atom were unrestrained during all energy minimisations (3,053 atoms), and all atoms further away were kept frozen. Calculations were performed using the ONIOM method in Gaussian16 rev. C.01^52^, using the B3LYP functional and 6–31 G(d,p) basis sets for all QM atoms and the Amber FF96 force field for the MM region. Electronic embedding was used for the electrostatic interaction between the MM and QM regions, the micro-iterations for optimizing the MM region were coupled to the quadratic macro steps for optimizing the QM region, and force constants were calculated during the initial step using Opt = (CalcFC, QuadMacro). Relaxed potential energy scans were performed as follows: for step 3 a reaction coordinate defined as the difference between the breaking and forming bonds, *z* = R(C-H) - R(O-H), was scanned with step size of 0.1 Å, and for step 4 the C-N bond was scanned with step size of 0.05 Å. Both steps were scanned multiple times, forwards and in reverse, until the energies converged. The highest-energy structures were then optimized to the transition states using Opt = (TS, CalcFC, QuadMacro). Zero-point energy corrections were calculated using frequency calculations, which were also used to confirm that transition states have one imaginary frequency and other stationary points have none.

### Supplementary information

Supplementary Information

### Source data

Source data

## Data Availability

The data generated in this study are provided within the paper and in the Supplementary Information. Source Data are provided with this paper. The coordinates and structure factors for the crystallographic data in this study are available in the Protein Data Bank under accession numbers 8BP1 and 8BP0 for BH_MeHis_1.0 and BH_MeHis_1.8, respectively. Source data are provided with this paper.

## References

[1] Lovelock SL (2022). The road to fully programmable protein catalysis. Nature.

[2] Hilvert D (2013). Design of protein catalysts. Annu. Rev. Biochem..

[3] Bolon DN, Mayo SL (2001). Enzyme-like proteins by computational design. Proc. Natl Acad. Sci. USA.

[4] Zanghellini A (2006). New algorithms and an in silico benchmark for computational enzyme design. Protein Sci..

[5] Kiss G, Çelebi-Ölçüm N, Moretti R, Baker D, Houk KN (2013). Computational enzyme design. Angew. Chem. Int. Ed..

[6] Siegel JB (2010). Computational design of an enzyme catalyst for a stereoselective bimolecular diels-alder reaction. Science.

[7] Privett HK (2012). Iterative approach to computational enzyme design. Proc. Natl Acad. Sci. USA.

[8] Röthlisberger D (2008). Kemp elimination catalysts by computational enzyme design. Nature.

[9] Jiang L (2008). De novo computational design of retro-aldol enzymes. Science.

[10] Blomberg R (2013). Precision is essential for efficient catalysis in an evolved Kemp eliminase. Nature.

[11] Preiswerk N (2014). Impact of scaffold rigidity on the design and evolution of an artificial Diels-Alderase. Proc. Natl Acad. Sci. USA.

[12] Obexer R (2017). Emergence of a catalytic tetrad during evolution of a highly active artificial aldolase. Nat. Chem..

[13] Crawshaw R (2022). Engineering an efficient and enantioselective enzyme for the Morita–Baylis–Hillman reaction. Nat. Chem..

[14] Giger L (2013). Evolution of a designed retro-aldolase leads to complete active site remodeling. Nat. Chem. Biol..

[15] Zhao J, Burke AJ, Green AP (2020). Enzymes with noncanonical amino acids. Curr. Opin. Chem. Biol..

[16] Birch-Price Z, Taylor CJ, Ortmayer M, Green AP (2023). Engineering enzyme activity using an expanded amino acid alphabet. Protein Eng. Des. Sel..

[17] Drienovská I, Mayer C, Dulson C, Roelfes G (2018). A designer enzyme for hydrazone and oxime formation featuring an unnatural catalytic aniline residue. Nat. Chem..

[18] Trimble JS (2022). A designed photoenzyme for enantioselective [2+2]-cycloadditions. Nature.

[19] Sun N (2022). Enantioselective [2+2]-cycloadditions with triplet photoenzymes. Nature.

[20] Burke AJ (2019). Design and evolution of an enzyme with a non-canonical organocatalytic mechanism. Nature.

[21] Green AP, Hayashi T, Mittel PRE, Hilvert D (2016). A chemically programmed proximal ligand enhances the catalytic properties of a heme enzyme. J. Am. Chem. Soc..

[22] Richter F (2012). Computational design of catalytic dyads and oxyanion holes for ester hydrolysis. J. Am. Chem. Soc..

[23] Burton AJ, Thomson AR, Dawson WM, Brady RL, Woolfson DN (2016). Installing hydrolytic activity into a completely de novo protein framework. Nat. Chem..

[24] Wurz RP (2007). Chiral dialkylaminopyridine catalysts in asymmetric synthesis. Chem. Rev..

[25] O’Reilly E (2022). Building enzymes from scratch. Nat. Chem..

[26] Wei Y, Shi M (2013). Recent advances in organocatalytic asymmetric Morita-Baylis-Hillman/aza- Morita-Baylis-Hillman reactions. Chem. Rev..

[27] Basavaiah D, Rao AJ, Satyanarayana T (2003). Recent advances in the Baylis-Hillman reaction and applications. Chem. Rev..

[28] Basavaiah D, Reddy BS, Badsara SS (2010). Recent contributions from the Baylis-Hillman reaction to organic chemistry. Chem. Rev..

[29] Bjelic S (2013). Computational design of enone-binding proteins with catalytic activity for the Morita-Baylis-Hillman reaction. ACS Chem. Biol..

[30] Xiao H (2014). Genetic incorporation of histidine derivatives using an engineered pyrrolysyl-tRNA synthetase. ACS Chem. Biol..

[31] Olsson MH, Søndergaard CR, Rostkowski M, Jensen JH (2011). PROPKA3: consistent treatment of internal and surface residues in empirical pKa predictions. J. Chem. Theory Comput..

[32] Bryson D (2017). Continuous directed evolution of aminoacyl-tRNA synthetases. Nat. Chem. Biol..

[33] Mehl RA (2003). Generation of a bacterium with a 21 amino Acid. Genet. Code J. Am. Chem. Soc..

[34] Wannier TM, Kunjapur AM, Rice DP, Church GM (2018). Adaptive evolution of genomically recoded Escherichia coli. Proc. Natl Acad. Sci. USA.

[35] Hossack EJ, Hardy FJ, Green AP (2023). Building enzymes through design and evolution. ACS Catal..

[36] Lee TS (2011). BglBrick vectors and datasheets: a synthetic biology platform for gene expression. J. Biol. Eng..

[37] Kille S (2012). Reducing codon redundancy and screening effort of combinatorial protein libraries created by saturation mutagenesis. ACS Synth. Biol..

[38] Luo S, Wang PG, Cheng JP (2003). Remarkable rate acceleration of imidazole-promoted Baylis-Hillman reaction involving cyclic enones in basic water solution. J. Org. Chem..

[39] Kataoka T, Iwama T, Tsujiyama S, Iwamura T, Watanabe S (1998). The chalcogeno-Baylis-Hillman reaction: a new preparation of allylie alcohols from aldehydes and electron-deficient alkenes. Tetrahedron.

[40] Shi M, Liu XG (2008). Asymmetric Morita-Baylis-Hillman reaction of arylaldehydes with 2-cyclohexen-1-one catalyzed by chiral bis(thio)urea and DABCO. Org. Lett..

[41] Aggarwal VK, Emme I, Fulford SY (2003). Correlation between pKa and reactivity of quinuclidine-based catalysts in the Baylis-Hillman reaction: discovery of quinuclidine as optimum catalyst leading to substantial enhancement of scope. J. Org. Chem..

[42] Li G, Wei H, Gao JJ, Caputo TD (2000). TiCl4-mediated Baylis-Hillman and aldol reactions without the direct use of a Lewis base. Tetrahedron.

[43] Vazquez-chavez J (2019). The effect of chiral N-substituents with methyl or trifluoromethyl groups on the catalytic performance of mono- and bifunctional thioureas. Org. Biomol. Chem..

[44] Wang F (2011). A highly efficient kinetic resolution of Morita-Baylis-Hillman adducts achieved by N-Ar axially chiral Pd-complexes catalyzed asymmetric allylation. Chem. Commun..

[45] Li K, Alexakis A (2006). Asymmetric conjugate addition to α-halo enones: dramatic effect of styrene on the enantioselectivity. Angew. Chem. Int. Ed..

[46] Zhang J (2011). Total synthesis of malyngamides K, L, and 5”-epi-C and absolute configuration of malyngamide L. J. Org. Chem..

[47] Baldwin JE, Adlington RM, Robertson J (1988). Carbocyclic ring expansion reactions via radical chain processes. J. Chem. Soc. Chem. Commun..

[48] Adams PD (2010). PHENIX: a comprehensive python-based system for macromolecular structure solution. Acta Cryst..

[49] Chen VB (2010). MolProbity: all-atom structure validation for macromolecular crystallography. Acta Crystallogr. D. Biol. Crystallogr..

[50] Joosten RP, Joosten K, Cohen SX, Vriend G, Perrakis A (2011). Automatic rebuilding and optimization of crystallographic structures in the protein data bank. Bioinformatics.

[51] Wang J, Wang W, Kollman PA, Case DA (2005). Antechamber: an accessory software package for molecular mechanical calculations. J. Chem. Comput. Chem..

[52] Frisch, M. J. et al. *Gaussian 16, Revision C.01* http://gaussian.com/citation (2016).

[53] Abraham MJ (2015). GROMACS: High performance molecular simulations through multi-level parallelism from laptops to supercomputers. SoftwareX.

[54] Páll, S., Abraham, M. J., Kutzner, C., Hess, B. & Lindahl, E. *Tackling Exascale Software Challenges in Molecular dynamics simulations with GROMACS.* https://digiedit3.mpslimited.com/Digicore/DigiEditPage.aspx?FileName=859609079171665119447256.xml (2015).

[55] Maier JA (2015). ff14SB: improving the accuracy of protein side chain and backbone parameters from ff99SB. J. Chem. Theory Comput..

[56] Bussi G, Donadio D, Parrinello M (2007). Canonical sampling through velocity rescaling. J. Chem. Phys..

[57] Nosé S, Klein ML (1983). Constant pressure molecular dynamics for molecular systems. Mol. Phys..

[58] Hess B, Bekker H, Berendsen HJC, Fraaije JGEM (1997). LINCS: a linear constraint solver for molecular simulations. J. Comput. Chem..

[59] Chai J-D, Head-Gordon M (2008). Long-range corrected hybrid density functionals with damped atom–atom dispersion corrections. Phys. Chem. y. Chem. Phys..

[60] Chai J-D, Head-Gordon M (2008). Systematic optimization of long-range corrected hybrid density functionals. J. Chem. Phys..

[61] Møller C, Plesset MS (1934). Note on an approximation treatment for many-electron systems. Phys. Rev..

[62] Boys SF, Bernardi FJMP (1970). The calculation of small molecular interactions by the differences of separate total energies. Some procedures with reduced errors. Mol. Phys..

