# Supplementary material for: A non-canonical nucleophile unlocks a new mechanistic pathway in a designed enzyme
Source: Nat Commun. 2024 Mar 4;15:1956. doi: 10.1038/s41467-024-46123-z (PMC10912507; doi:10.1038/s41467-024-46123-z)
Supplement: Supplementary file 1 — Supplementary Information [file 41467_2024_46123_MOESM1_ESM.pdf]

Supplementary Information

A Non-Canonical Nucleophile Unlocks a New Mechanistic Pathway in a Designed Enzyme

Amy E. Hutton[1], Jake Foster[1], Rebecca Crawshaw[1], Florence J. Hardy[1], Linus O. Johannissen[1], Thomas M. Lister[1], Emilie F. Gérard[1], Zachary Birch-Price[1], Richard Obexer[1], Sam Hay[1], Anthony P. Green[1]*

*Corresponding author

[1]Manchester Institute of Biotechnology, School of Chemistry, The University of Manchester, Manchester, UK.

Table of Contents:

Supplementary Figures 1-16 4
Supplementary Figure 1: Identification of a suitable starting point for evolution 4
Supplementary Figure 2: Comparison of pH profiles of BH32.8 and BHMeHis1.0 5
Supplementary Figure 3: UPLC analysis of the MBH reaction catalysed by either BHMeHis1.0 or BHMeHis1.8 6
Supplementary Figure 4: pH profiles of BHMeHis1.8 and BH32.14 7
Supplementary Figure 5: Kinetic characterization of BHMeHis1.0, BHMeHis1.8 and BHMeHis1.8 MeHis23His 8
Supplementary Figure 6: Total turnover numbers achieved by BHMeHis1.8 9
Supplementary Figure 7: Temperature profile of BHMeHis1.8........ 10
Supplementary Figure 8: NMR traces of crude reaction and purified **3** produced from a preparative-scale biotransformation 11
Supplementary Figure 9: Crystal structures of BHMeHis1.0 and BHMeHis1.8 12
Supplementary Figure 10: Structural parameters for MD simulation of BH_{MeHis}1.8 apo complex....... 13
Supplementary Figure 11: Models for calculation of Trp42 stabilisation........14
Supplementary Figure 12: Docking of MBH product 3 into the crystal structure of BHMeHis1.8 15
Supplementary Figure 13: Changes in reaction rate upon mutation of Glu26 in BHMeHis1.8 to either Gln or Ala 16
Supplementary Figure 14. Time course of the inhibition of MBH1.8 and its variants........ 17
Supplementary Figure 15: Representative MD snapshot of BHMeHis1.8:Int2H complex where the proton has been transferred from Glu(H)26 to Int2 (model B) from a 500 ns simulation 18
Supplementary Figure 16. Structural parameters for MD simulation of BHMeHis1.8:Int2 complex with a protonated glutamic acid (Glu(H)26) (model A) 19
Supplementary Figure 17. Structural parameters for MD simulation of BHMeHis1.8:Int2H complex where the proton has been transferred from Glu(H)26 to Int2 (model B)........ 20
Supplementary Figure 18: Computed reaction profile for BH_{MeHis}1.8 starting from Int2H........ 21
Supplementary Figure 19: QM/MM models along the BHMeHis1.8 reaction coordinate........ 22
Supplementary Figure 20: MD simulation showing the rapid rearrangement of product bound structure P from QM/MM........ 23
Supplementary Figure 21:Changes in activity along the evolutionary trajectory upon mutation of MeHis23 nucleophile to histidine 24
Supplementary Tables 1-9 ....
Supplementary Table 1: Directed evolution of BHMeHis1.8........ 25
Supplementary Table 2: Enantiomeric excess of BHMeHis1.0, BHMeHis1.8 and selected variants.... 26
Supplementary Table 3: Kinetic characterization of BHMeHis1.0, BHMeHis1.8 and BHMeHis1.8 MeHis23His 27

Supplementary Table 4: Conversions of MBH reactions catalysed by BHMeHis1.0, BHMeHis1.8 and selected variants 28

Supplementary Table 5: Effect of cosolvent on BHMeHis1.8 activity 29

Supplementary Table 6: Reaction conditions for the substate scope to synthesise 3 and 4a-k.......... 30

Supplementary Table 7: Data collection and refinement statistics 31

Supplementary Table 8: Kinetic isotope effect (KIE) and solvent kinetic isotope (SKIE) effects for BHMeHis1.8 and selected variants 32

Supplementary Table 9: Experimental and calculated masses of apo enzymes 33

Supplementary Table 10: Table of primers used in this study 34

Supplementary Table 11: Table of primers used for library generation 35

DNA and protein sequence for the most active variant $BH_{MeHis}1.8$........ 46

Supplementary Figures 1- 16

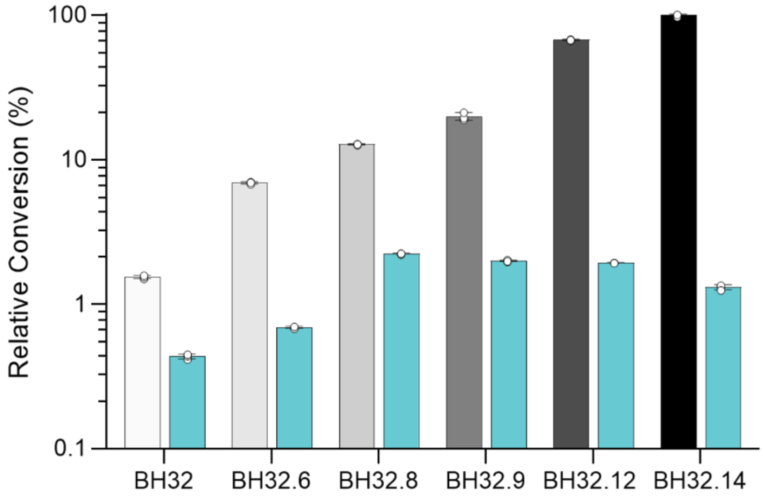

Supplementary Figure 1: Identification of a suitable starting point for evolution. Relative conversions of BH32[1] and selected evolved descendants[2] with either His (grey scale) or MeHis (blue) as the catalytic nucleophile at position 23. Biotransformations were performed using **1** (15 mM), **2** (1.5 mM) and enzyme (60 µM) in PBS (pH 7.4) with 3% (v/v) MeCN as cosolvent and analysed following 5 h incubation at 30 °C. Error bars represent the standard deviation of measurements made in triplicate. Source Data are provided as a Source Data file.

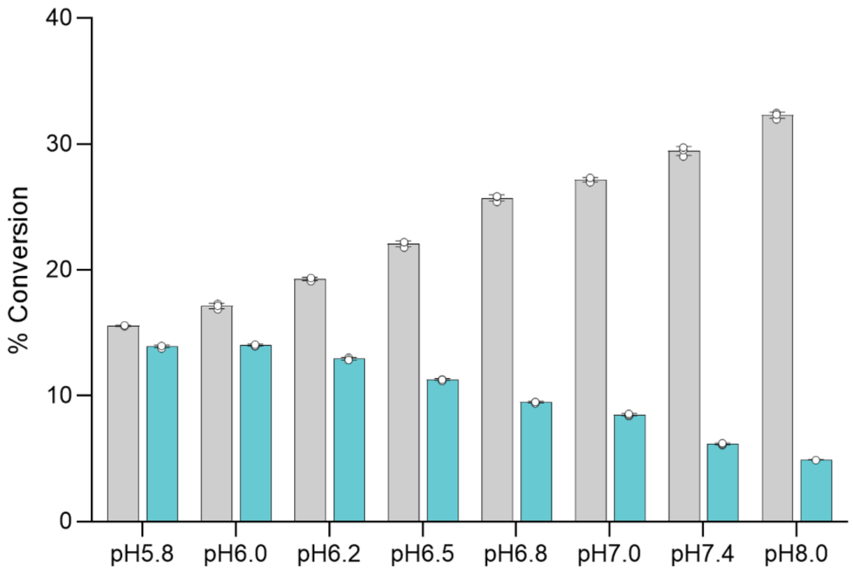

Supplementary Figure 2: Comparison of pH profiles of BH32.8 and BH$_{MeHis}$1.0. Reaction conversions of BH32.8[2] (grey) and BH$_{MeHis}$1.0 (blue) across a range of pHs ranging from pH 5.8 to 8.0. Reactions performed using **1** (15 mM), **2** (1.5 mM), 60 µM enzyme in PBS at the stated pH using 3% (v/v) MeCN as cosolvent and analysed following 21 h incubation at 30 °C. Error bars represent standard deviation of measurement made in triplicate. In addition to MBH product **3** an aldol side product (**S1**) was formed in low abundance (data not shown). Source Data are provided as a Source Data file.

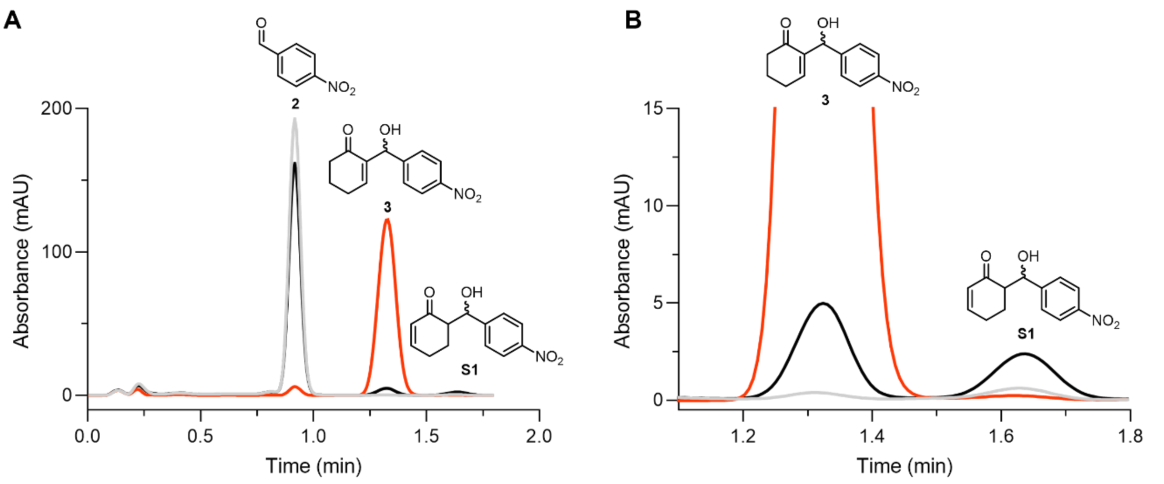

Supplementary Figure 3: UPLC analysis of the MBH reaction catalysed by either BH$_{MeHis}$1.0 or BH$_{MeHis}$1.8. A) Ultra-high performance liquid chromatography (UPLC) trace for the MBH reaction between 2-cyclohexen-1-one (**1**) and 4-nitrobenzaldehyde (**2**) forming MBH product (**3**) and an aldol side product (**S1**). **B)** An expanded view of the UPLC trace presented in **A** between 1.1 and 1.8 min. After evolution, BH$_{MeHis}$1.8 (red, 10 µM) forms MBH product **3** as the exclusive product as opposed to the starting variant BH$_{MeHis}$1.0 (grey, 10 µM or black, 60 µM). Reactions performed using **1** (15 mM), **2** (1.5 mM), PBS pH 6.0 with 20% (v/v) DMSO as cosolvent and analysed following 23 h incubation at 30 °C.

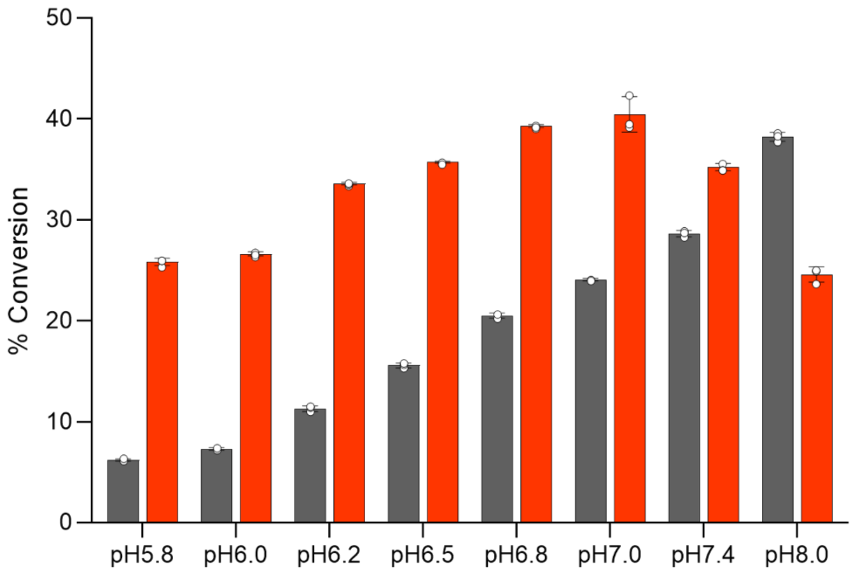

Supplementary Figure 4: pH profiles of BH$_{MeHis}$1.8 and BH32.14. Reaction conversions of either BH$_{MeHis}$1.8 (red, 3 µM) or BH32.14[2] (grey, 30 µM) achieved across a range of pHs between 5.8 to 8.0. Reactions were performed using **1** (15 mM), **2** (1.5 mM) in PBS at the stated pH using 3% (v/v) MeCN as cosolvent and analysed by UPLC following 2 h incubation at 30 °C. Error bars represent standard deviation of measurement made in triplicate. Source Data are provided as a Source Data file.

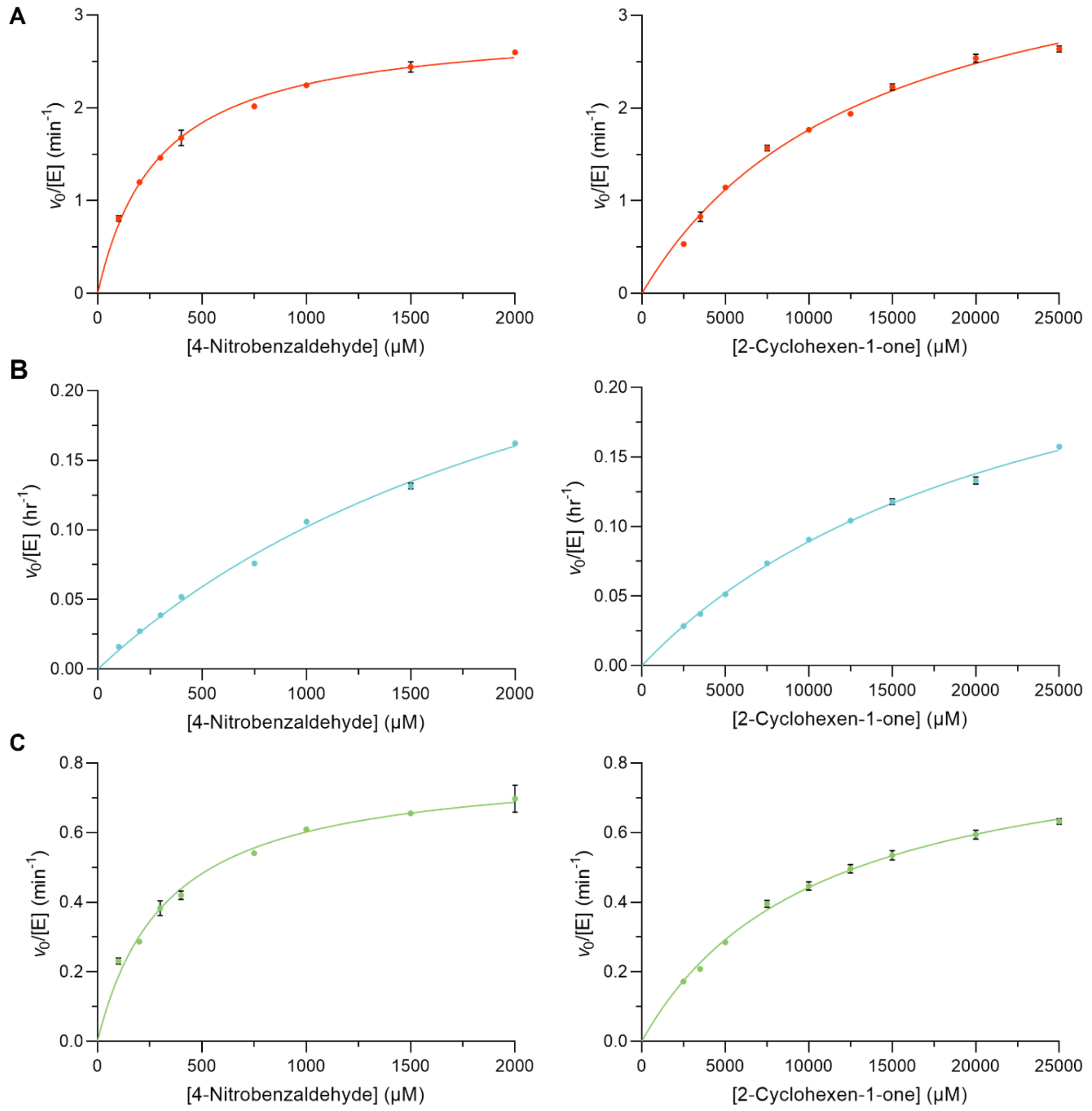

Supplementary Figure 5: Kinetic characterization of BH$_{MeHis}$1.0, BH$_{MeHis}$1.8 and BH$_{MeHis}$1.8 MeHis23His. Michaelis-Menten plots for the MBH reaction between **1** and **2** catalysed by either: **A)** BH$_{MeHis}$1.8, **B)** BH$_{MeHis}$1.0 and **C)** BH$_{MeHis}$1.8 MeHis23His. Assays were performed at either a fixed concentration of **1** (25 mM) and varying concentrations of **2**, or a fixed concentration of **2** (2 mM) and varying concentrations of **1**. The plots show the averaged initial rates of triplicate data, along with error bars, which were fitted to the Michaelis-Menten equation using Origin software. Source Data are provided as a Source Data file.

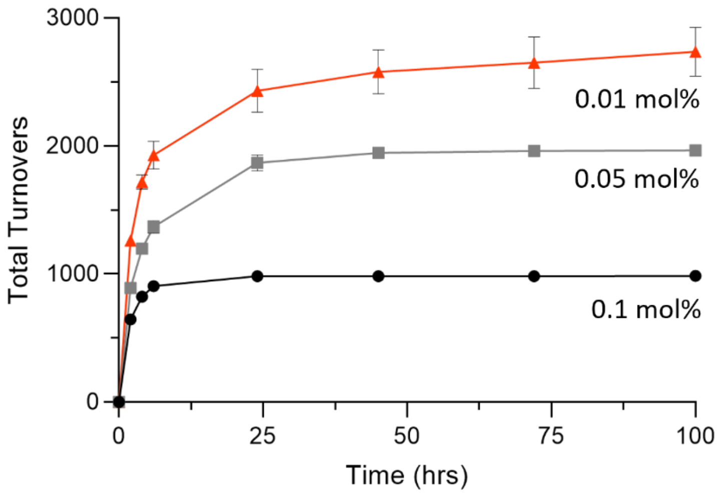

Supplementary Figure 6: Total turnover numbers achieved by BH$_{MeHis}$1.8. Time-course to determine the total turnover number of BH$_{MeHis}$1.8. Reactions were performed using **1** (50 mM) and **2** (10 mM) in PBS pH 7.0 with 20% (v/v) DMSO as cosolvent, using either 0.1 mol% (black), 0.05 mol% (grey) or 0.01 mol% (red) of BH$_{MeHis}$1.8. Error bars represent standard deviation of measurement made in triplicate. Source Data are provided as a Source Data file.

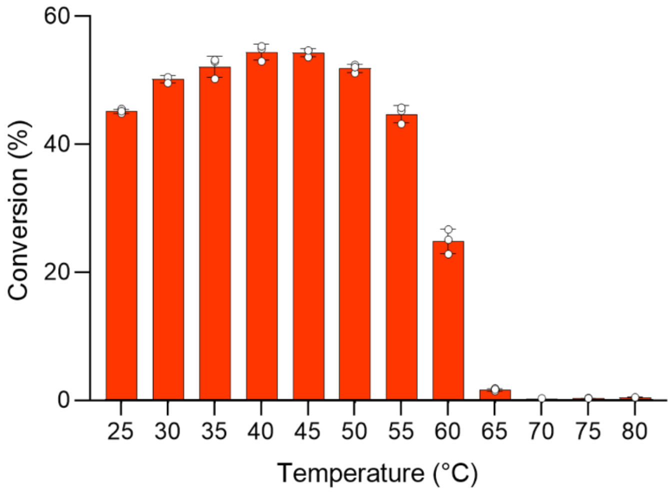

Supplementary Figure 7: Temperature profile of BH$_{MeHis}$1.8. Reaction conversions of BH$_{MeHis}$1.8 across a range of temperatures from 25 to 80 °C after 2 h incubation. Reactions were performed using **1** (15 mM), **2** (1.5 mM) in PBS pH 7.0 using 3% (v/v) MeCN as cosolvent and 3 µM BH$_{MeHis}$1.8. Error bars represent standard deviation of measurement made in triplicate. Aldol by-product (**S1**) only observed at temperatures above 60 °C (data not shown). Source Data are provided as a Source Data file.

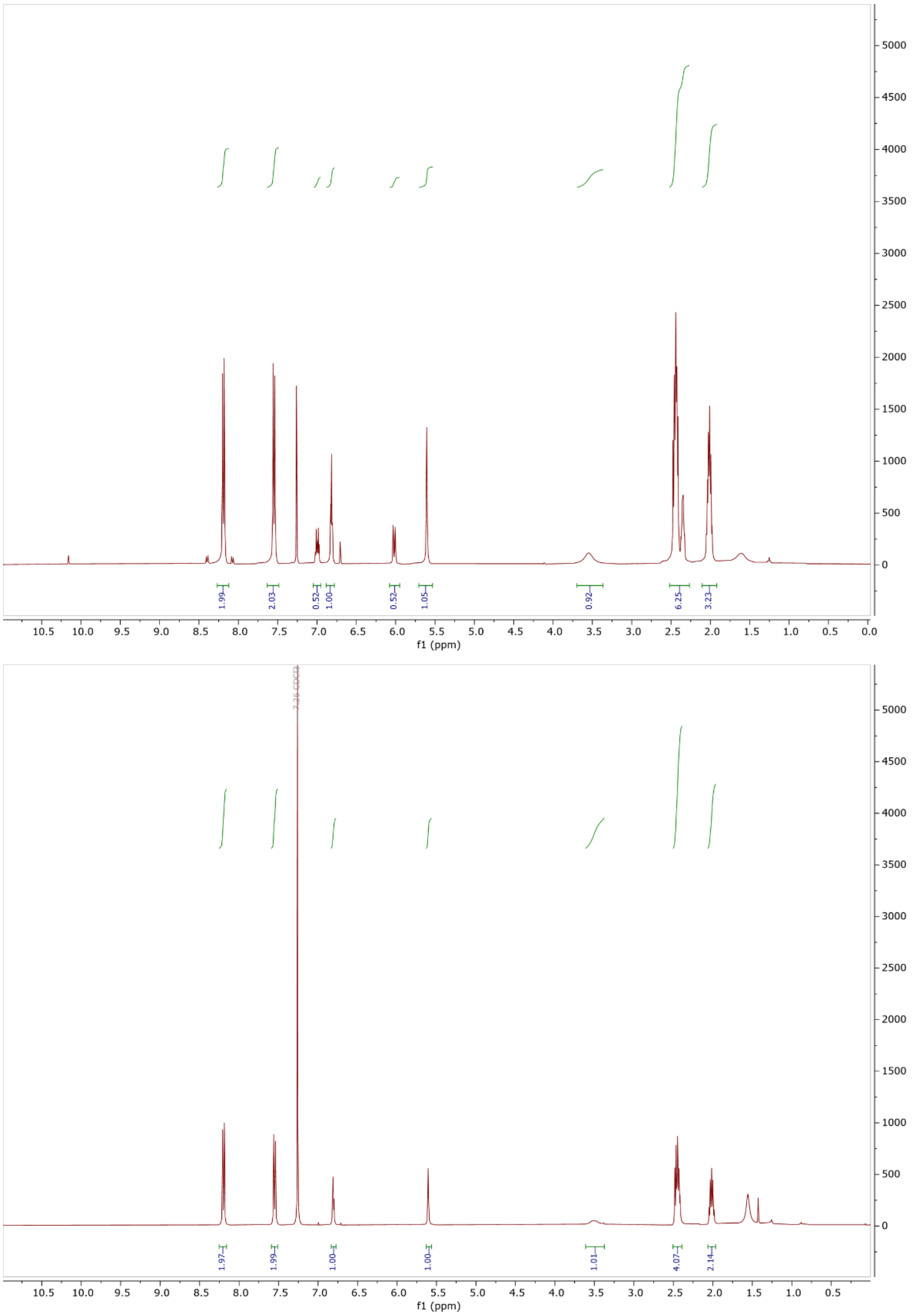

Supplementary Figure 8: NMR traces of crude reaction and purified 3 produced from a preparative-scale biotransformation. ^{1}H NMR traces (400 MHz; $CDCl_3$) showing; Top: crude product extracted from the preparative-scale biotransformation of BH_{MeHis}1.8 (10 μM) using **1** (50 mM) and **2** (10 mM) in PBS pH 7.0 with 20% (v/v) DMSO as cosolvent for 13 h at 30 °C. Bottom: Isolated MBH product **3** following purification by flash chromatography. Spectral data is consistent with literature values.[3]

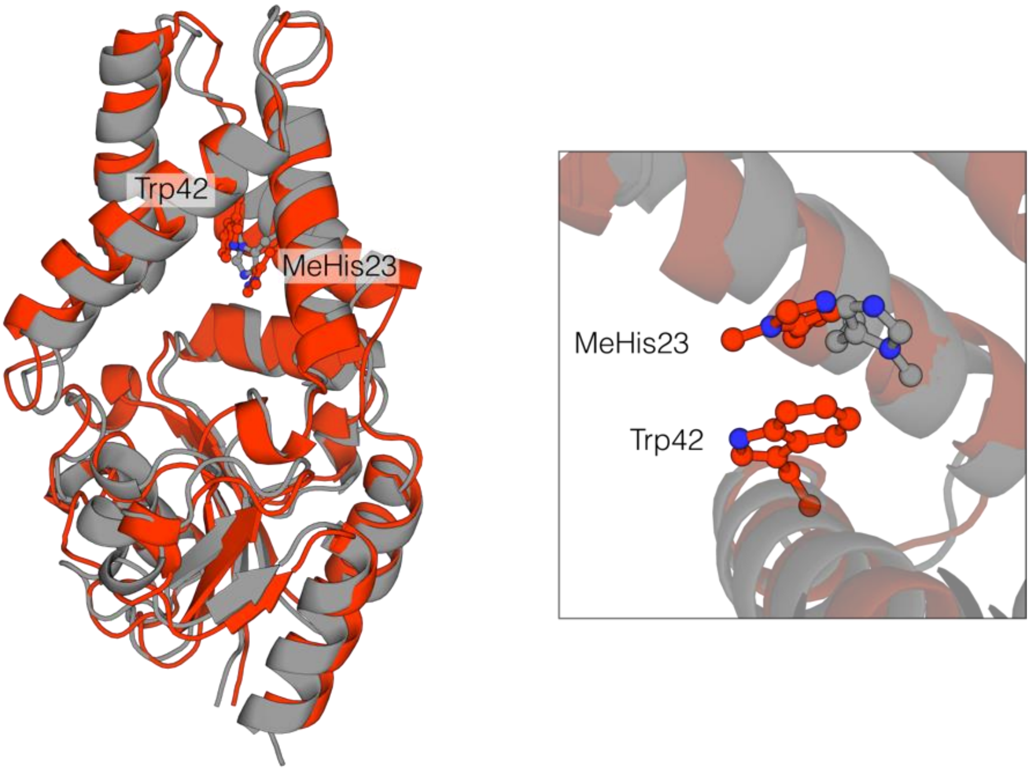

Supplementary Figure 9: Crystal structures of BH$_{MeHis}$1.0 and BH$_{MeHis}$1.8. A cartoon presentation (left) of the superimposed coordinates of BH$_{MeHis}$1.0 (grey) and BH$_{MeHis}$1.8 (red). Mutations installed during evolution cause minimal changes to the overall protein fold, with a secondary structure root mean square deviation of 0.47 Å. The MeHis23 nucleophile is shown as atom coloured ball and sticks in both structures, and Trp42 is shown in BH$_{MeHis}$1.8. A zoom of the active site (right) shows a ~120° rotation in the imidazole ring has occurred during evolution.

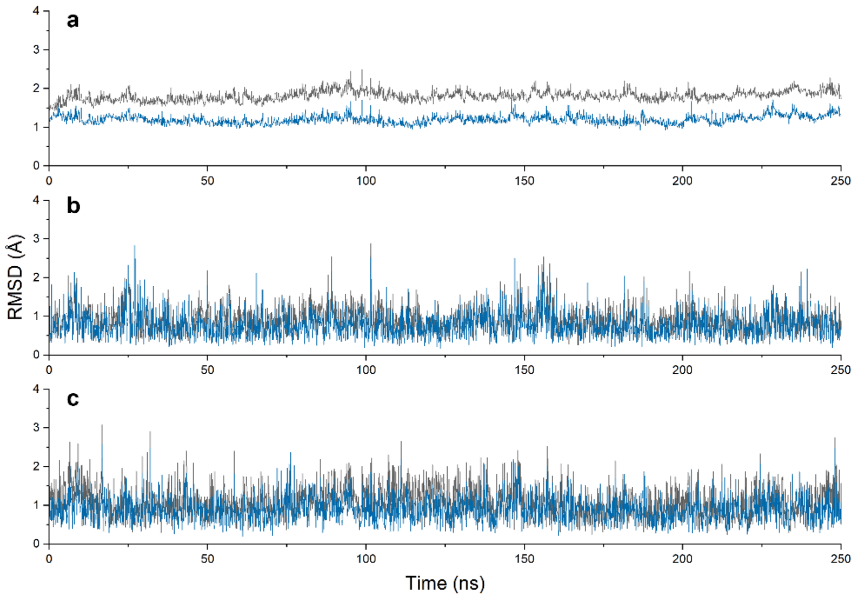

Supplementary Figure 10: Structural parameters for MD simulation of BH_{MeHis}1.8 apo complex. A) protein heavy-atom rmsd (main and side chain atoms) relative to the first (grey) and average (blue) structure. **B**) MeHis23 rmsd relative to the first (grey) and average (blue) structure. **C**) Trp42 rmsd relative to the first (grey) and average (blue) structure.

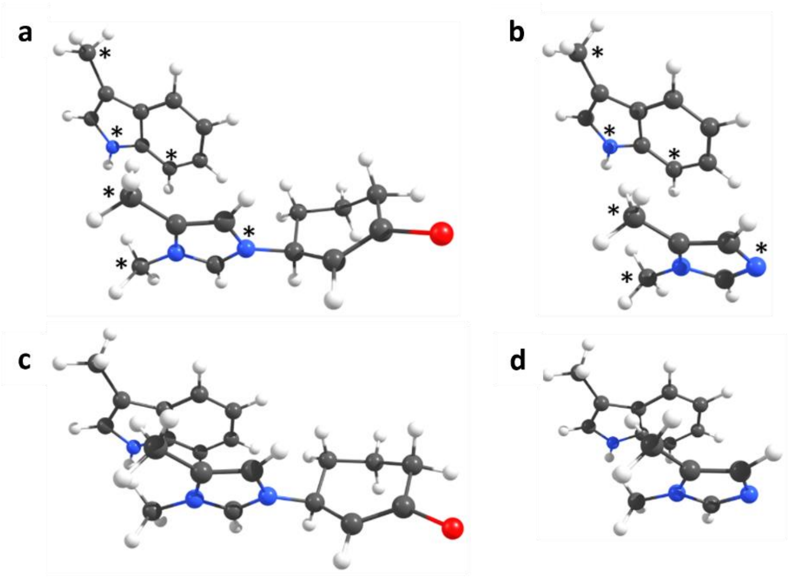

e

Model of position 42	ΔΔE (kJ mol^{-1})	
	DFT	MP2
Methyl indole (constrained) **b**	-5.0	-5.1
Methyl indole (unconstrained) **d**	-12.2	-8.3
Toluene (unconstrained)	-8.7	-5.7

Supplementary Figure 11: Models for calculation of Trp42 stabilisation. For simplicity only models with methyl indole for calculating ΔE_2 (equation 2) are shown. **A** and **C**) Int1 models. **B** and **D**) MeHis models. * indicates atom kept fixed during energy minimisation in the constrained models **A** and **B**. **E**) Table of ΔΔE energies calculated using either DFT or MP2 for models **B** and **D** along with the energies calculated for a Trp42Phe mutation with Phe modelled as toluene.

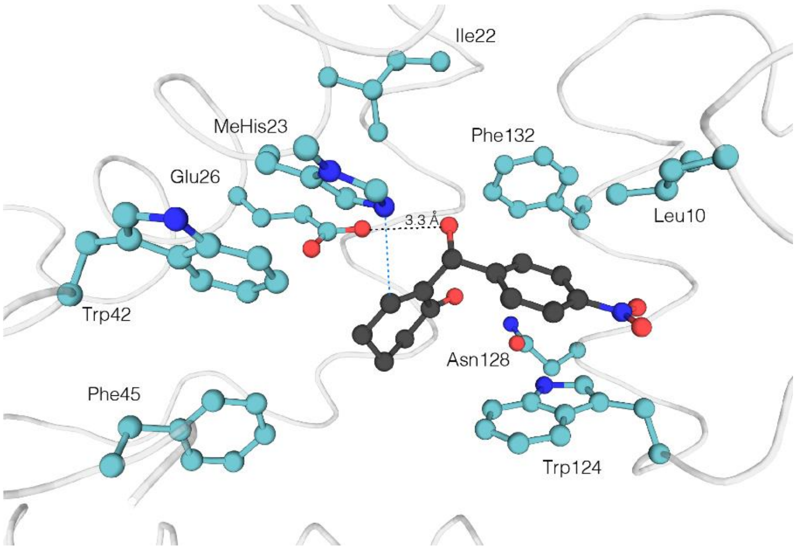

Supplementary Figure 12: Docking of MBH product 3 into the crystal structure of BH$_{MeHis}$1.8. The product (*R*)-**3** (shown as atom-coloured sticks, carbons black) was docked into the crystal structure of BH$_{MeHis}$1.8 using MolsoftICM64-Pro (version 3.9-2d). To ensure a productive pose for catalysis, a distance restraint of 4 Å between the MeHis and the position of nucleophilic attack was imposed on the calculation (shown as a blue dashed line). Glu26 is within hydrogen-bonding distance of O1 of **3** (black dashed line).

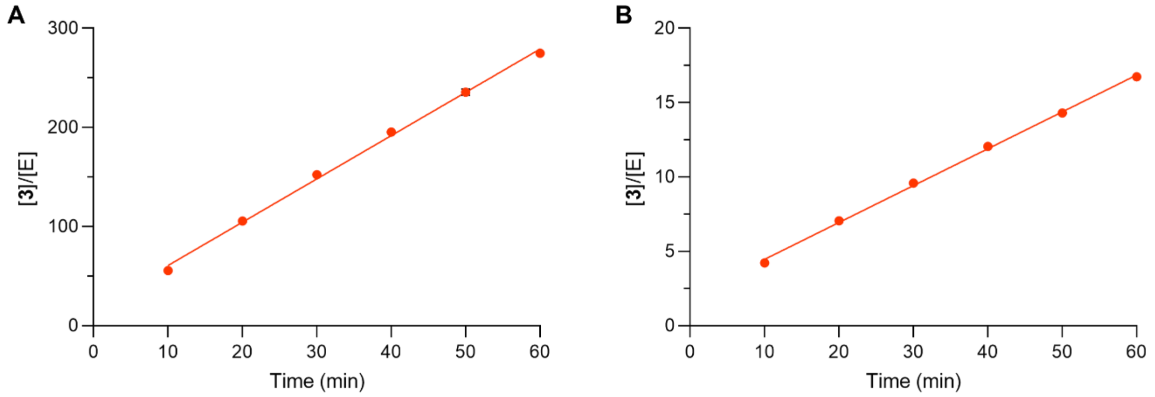

C

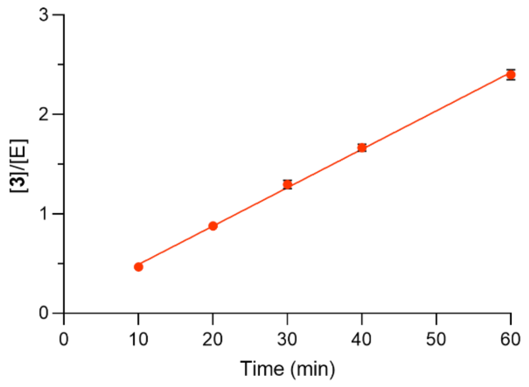

D

Variant	Rate (min^{-1})	S.D.
BH_{MeHis}1.8	4.367	0.059
BH_{MeHis}1.8 Glu26Gln	0.2479	0.002
BH_{MeHis}1.8 Glu26Ala	0.03864	0.006

Supplementary Figure 13: Changes in reaction rate upon mutation of Glu26 in BH_{MeHis}1.8 to either Gln or Ala. Reactions were performed using **1** (25 mM), **2** (2 mM) in PBS pH 7.0 with 3% (v/v) MeCN as cosolvent. **A)** BH_{MeHis}1.8, **B)** BH_{MeHis}1.8 Glu26Gln and **C)** BH_{MeHis}1.8 Glu26Ala. Error bars represent standard deviation of measurement made in triplicate. **D)** Table of rates determined from linear plots with standard deviation of triplicate measurements stated. Source Data are provided as a Source Data file.

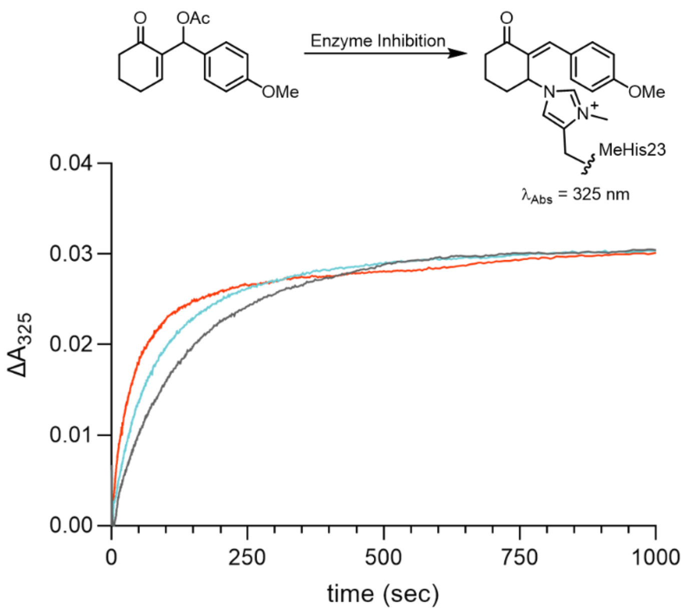

Supplementary Figure 14. Time course of the inhibition of BH$_{MeHis}$1.8 and its variants. Stopped flow analysis of mechanistic inhibitor binding,[2] through absorbance measurements at 325 nm to BH$_{MeHis}$1.8 (red), BH$_{MeHis}$1.8 Glu26Ala (blue) and BH$_{MeHis}$1.8 MeHis23His (grey). Reactions were performed using (*p*-methoxyphenyl)(6-oxocyclohex-1-en-1-yl)methyl acetate inhibitor (25µM) in PBS pH 7.0 with 3% (v/v) acetonitrile as cosolvent and 10 µM enzyme at room temperature. Source Data are provided as a Source Data file.

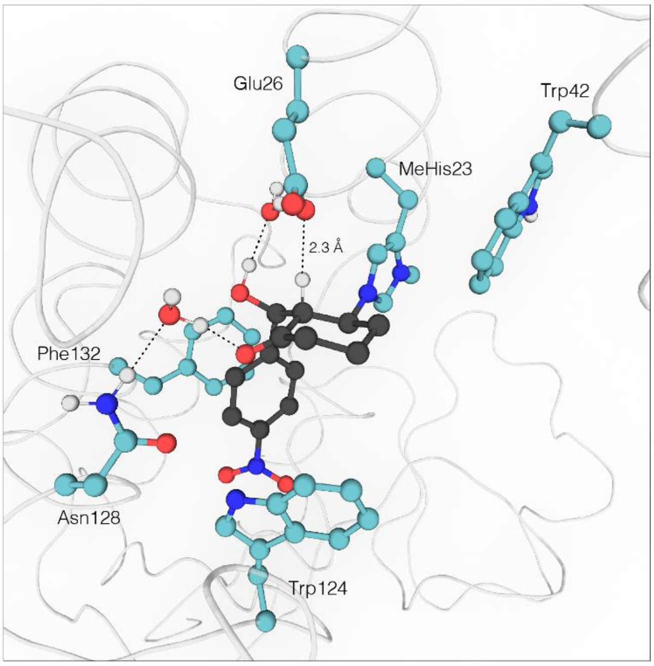

Supplementary Figure 15: Representative MD snapshot of BH$_{MeHis}$1.8:Int2H complex where the proton has been transferred from Glu(H)26 to Int2 (model B) from a 500 ns simulation. **Int2** (black) and key amino acid residues (blue) are shown in ball and stick representation with hydrogen bonds shown as black dashed lines.

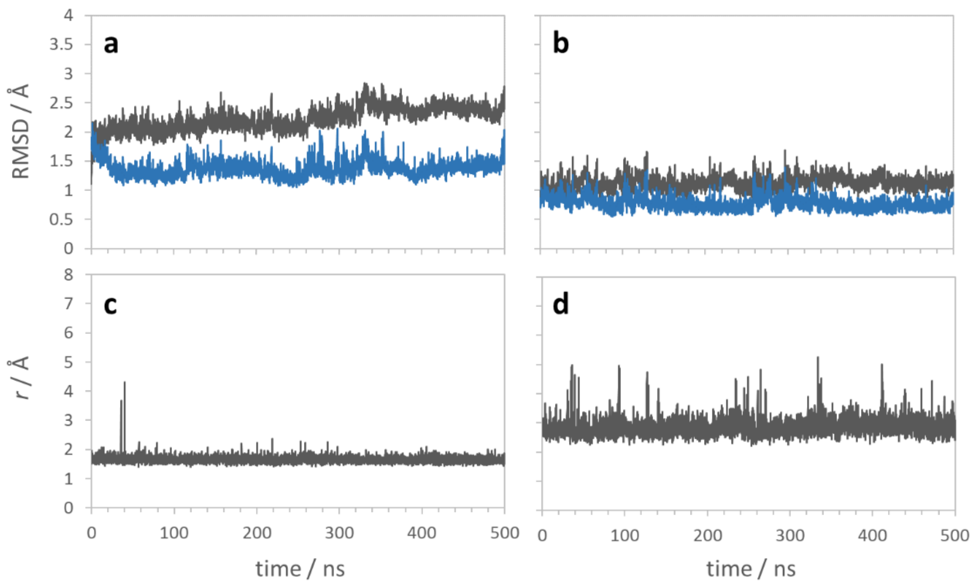

Supplementary Figure 16. Structural parameters for MD simulation of BH$_{MeHis}$1.8:Int2 complex with a protonated glutamic acid (Glu(H)26) (model A). (**a**) protein heavy-atom RMSD (main and side chain atoms) relative to the first (grey) and average (blue) structure, (**b**) active site RMSD relative to the first (grey) and average (blue) structure. The active site is defined as residues with at least one atom within 5 Å of the MeHis23_**Int2** adduct in the starting structure (residues number 10, 11, 14, 18, 19, 20, 21, 22, 23, 24, 25, 26, 27, 42, 45, 49, 68, 91, 92, 94, 122, 124, 128, 132). (**c**) O-H distance between the putative proton donor Glu(H)26 and the C3-alkoxide of **Int2**. (**d**) O-H distance between the C2 proton and the GluH26 carbonyl oxygen.

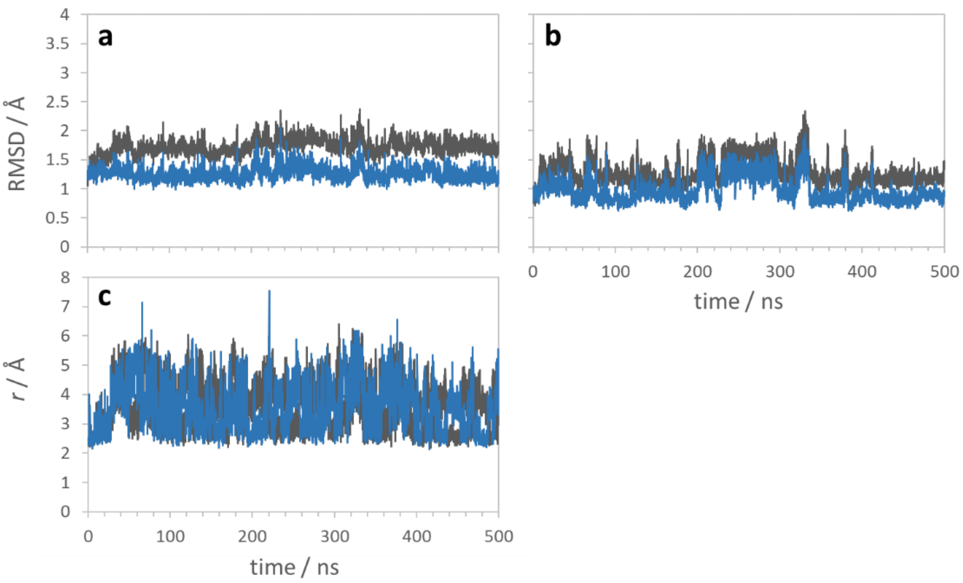

Supplementary Figure 17. Structural parameters for MD simulation of BH$_{MeHis}$1.8:Int2H complex where the proton has been transferred from Glu(H)26 to Int2 (model B). (**a**) protein heavy-atom RMSD (main and side chain atoms) relative to the first (grey) and average (blue) structure, (**b**) active site RMSD relative to the first (grey) and average (blue) structure. The active site is defined by the same residues as for model A (Supplementary Figure 14). (**c**) O-H distances between the C2-proton and each of the two oxygen atoms of Glu26 (one atom in blue and one in grey).

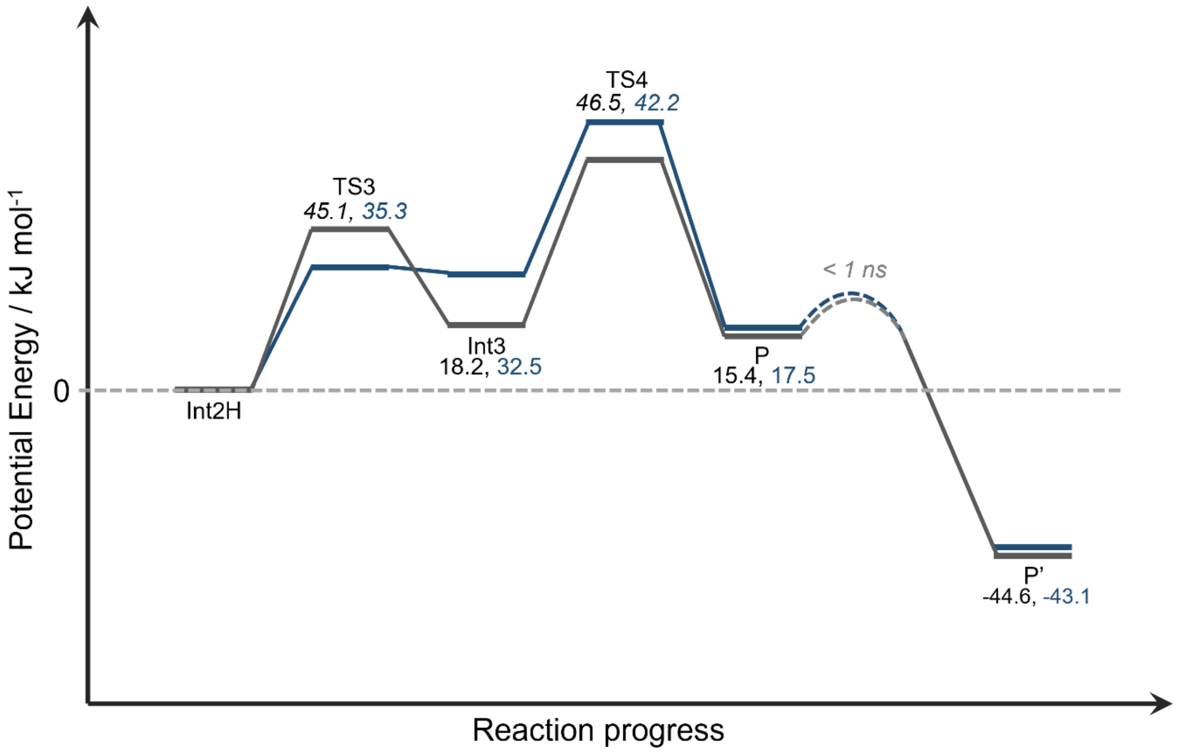

Supplementary Figure 18. Computed reaction profile for BH_{MeHis}1.8 starting from Int2H. The potential energies (black) and zero-point energy corrected energies (blue). The potential energies relative to Int2H are shown for each intermediate, with the barrier height for the transition state of that step (italics). The dotted line connecting P and P' indicates a reorganisation of the product state which occur on the sub-ns scale in MD simulations.

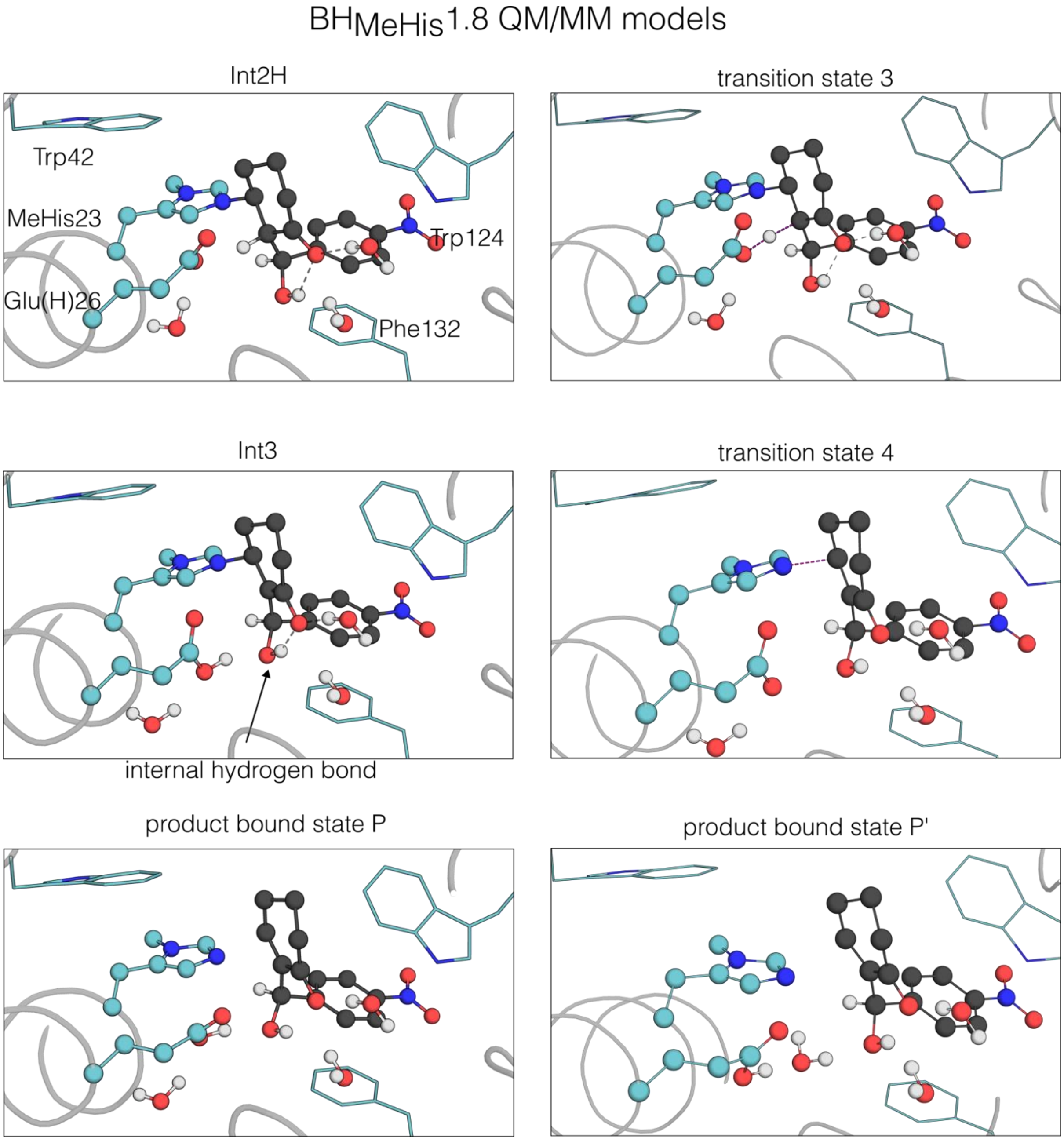

Supplementary Figure 19. QM/MM models along the BH$_{MeHis}$1.8 reaction coordinate. Transition states and intermediates along the reaction coordinate are shown. The protein backbone is shown as a grey ribbon. Trp42, Trp124, and Phe132 are shown as atom-coloured sticks, with light blue carbon atoms. Key residues, MeHis23 and Glu(H)26, and substrates, 2-cyclohexen-1-one and *p*-nitrobenzaldehyde are shown in atom coloured ball and stick representation with light blue and grey carbon atoms, respectively. Selected hydrogen atoms and water molecules are shown to aid mechanistic understanding.

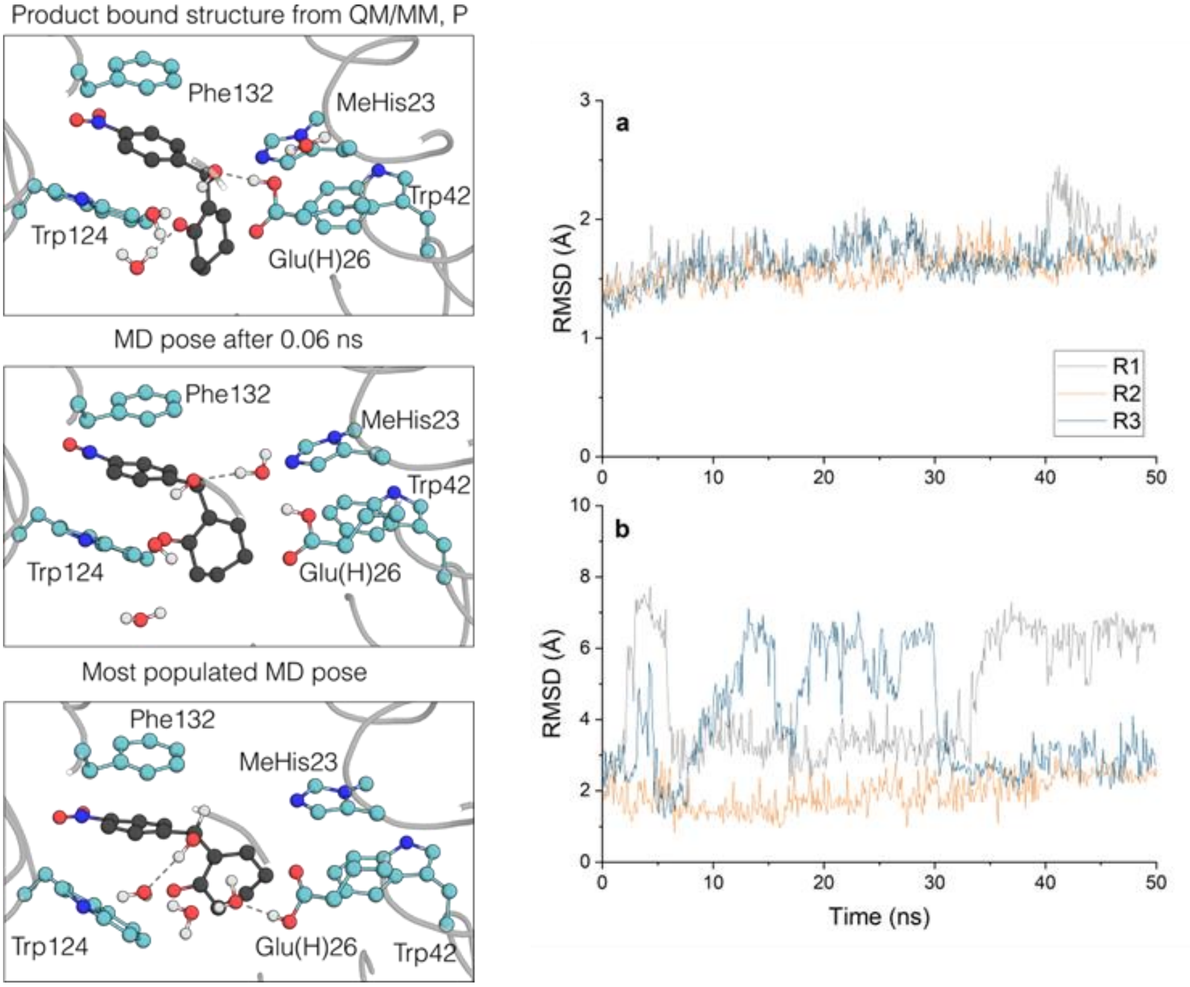

Supplementary Figure 20. MD simulation showing the rapid rearrangement of product bound structure P from QM/MM. Left: The optimized product bound structure from QM/MM, P and a MD frame of the $BH_{MeHis}1.8$ product complex after 0.06 ns and the MD structure representing the most populated pose over three 50 ns runs. Right: Structural parameters for the MD simulation of $BH_{MeHis}1.8$ product complex. **A)** protein heavy-atom RMSD (main and side chain atoms) relative to the first structure of Run 1 (grey), Run 2 (orange) and Run 3 (blue). **B)** Product RMSD relative to the first structure of Run 1 (grey), Run 2 (orange) and Run 3 (blue).

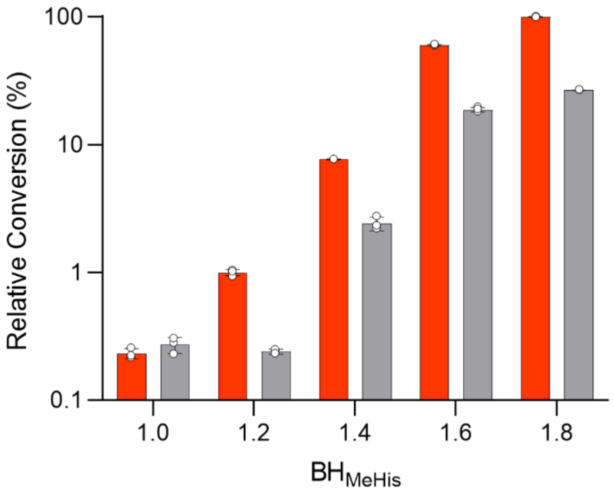

Supplementary Figure 21: Changes in activity along the evolutionary trajectory upon mutation of MeHis23 nucleophile to histidine. Relative conversions of variants along the evolutionary trajectory of BH_{MeHis}1.8 with either MeHis (red) or His (grey) as the catalytic nucleophile at position 23. Biotransformations were performed using **1** (15 mM), **2** (1.5 mM) and enzyme (1.5 µM) in PBS pH 6.0 with 3% (v/v) MeCN as cosolvent and analysed following 3 h incubation at 30 °C. Error bars represent the standard deviation of measurements made in triplicate. To eliminate errors arising from determination of low conversions, variants BH_{MeHis}1.0, BH_{MeHis}1.0 MeHis23His and BH_{MeHis}1.2 MeHis23His were monitored over a longer timeframe and conversions were extrapolated using linear regression. Source Data are provided as a Source Data file.

1. Supplementary Tables 1-9

Supplementary Table 1: Directed evolution of BH_{MeHis}1.8. Strategy employed for each round of evolution using either random mutagenesis or site-saturation mutagenesis, stating mutations introduced in each round. Starting template (BH_{MeHis}1.0) originated from our previous MBH evolution[2] where His23 has been mutated to MeHis.

Round	Description	Clones screened	Beneficial mutations	Best variant[1]
1	**Saturation mutagenesis** of positions mutated during BH32.14 evolution – L10, A19, Y20, V22, L24, I26, M27, L42, Y45, E46, Y56, L64, E70, D125, P128, A129, F132, F154, Y177, D180	1760	I26E, I26T, M27S, L42S, Y45R, L64A, P128F	**BH_{MeHis}1.1** = *BH_{MeHis}1.0*[2] + I26E_L64A
2	**Combinatorial active site saturation testing (CASTing)** of two 'hotspots' L42 and Y45 simultaneously randomised	1408	L42E/Y45R, L42V/Y45V, L42W/Y45F, L42Y/Y45F	**BH_{MeHis}1.2** = *BH_{MeHis}1.1* + L42W_Y45F
3	**Saturation mutagenesis** of active site positions and flexible loop regions – S9, I14, A19, Y20, V22, L24, M27, A49, F53, L87, W88, S91, L92, A95, N123, D125, P128, A131, F132, K174	1760	L24V, W88K, W88Q, S91G	**BH_{MeHis}1.3** = *BH_{MeHis}1.2*+ L24V_W88Q_S91G
4	**Random mutagenesis** of the entire gene	1936	R124W/E173G/G32A, T146S/E51D, V71I/Y177F, V71A/I215N/D66G, K74N/F161L, Q96R/K139R/D185G, E46D/K202N/K184E, E46D/L47Q, F161I/K202I/S217T	
5	**Saturation mutagenesis** of positions identified from random mutagenesis – G32, E46, L48, E51, D66, V71, K74, E85, Q96, R124, K139, T146, F161, E173, Y177, V178, K184, D185, K202, I215, S217, I223	1936	Q96R, R124W, F161L, Y177V	**BH_{MeHis}1.4** = *BH_{MeHis}1.3*+ Q96R_R124W_F161L
6	**Saturation mutagenesis** of 'hotspots' – V22, M27, E46, F53, Y56, D66, E85, L92, A95, D125 P128, F132, F154, E173, K174, Y177, I215 and active site positions – Y34, K39, L41, K110, Y116, L122, K155, A175, V178, E207, A212	1936	E46D, Y56G, D66R, E85G, L92V, P128N, P128W, F154A	**BH_{MeHis}1.5** = *BH_{MeHis}1.4* + E46D_E85G_L92V_ P128N_F154A
7	**Saturation mutagenesis** of active site positions and flexible loops – S9, L10, V22, Y34, K39, L41, W42, A49, F53, Y56, D66, E85, G91, A95, L122, D125, E127, N128, F154, K155, Y177, I215	1936	V22I, V22L, A49F, I215R	**BH_{MeHis}1.6** = *BH_{MeHis}1.5* + V22I_A49F_ I215R
8	**Random mutagenesis** of entire gene	1936	R26H/L165M, E100G, Y34H/S189T, L122M, S15T/K39N/E100G, D43N/E100G/S189P, R62H/D227A/N191I/ G193D, E100G/R220H, D66Y, S15T/E173V, K115E, V176A	
9	**Saturation mutagenesis** of positions identified from random mutagenesis – S15, E26, M27, Y34, K39, D43, R62, D66, E100, L112, K115, L122, N123, D125, L165, E173, V176, S189, N191, G193, R220, D227	1936	Y34H, Y34S, K39R, D66F, D66Y, E100G	**BH_{MeHis}1.7** = *BH_{MeHis}1.6* + K39R_D66F_E100G
10	**Saturation mutagenesis** of active site positions and flexible loop regions – A20, R50, E51, Y56, L68, L87, M94, A95, R97, Y98, G99, L101, Y102, T126, A129, F132, T146, K155, A175, Y177, E207, A212	1936	R50K, G99P, T146V, K155M, K155Y	**BH_{MeHis}1.8** = *BH_{MeHis}1.7*+ R50K_K155Y

[1] The gene sequences used as the template each round of evolution shown in italics
[2] BH_{MeHis}1.0 = BH32.8 with His23MeHis mutation

Supplementary Table 2: Enantiomeric excess of BH_{MeHis}1.0, BH_{MeHis}1.8 and selected variants. Reactions were performed using **1** (15 mM), **2** (1.5 mM), PBS pH 6.0 with 20% (v/v) DMSO as cosolvent and analysed by UPLC following 23 h incubation at 30 °C. All reactions ran with 10 µM enzyme apart from BH_{MeHis}1.0 (60 µM).

[a] Preparative-scale biotransformation. Performed using enzyme (10 µM), **1** (50 mM), **2** (10 mM), PBS pH 7.0 with 20% (v/v) DMSO as cosolvent.

**Variant**	***e.e.*(%)**
BH_{MeHis}1.0	55
BH_{MeHis}1.8	90
BH_{MeHis}1.8 MeHis23His	89
BH_{MeHis}1.8 Glu26Gln	97
BH_{MeHis}1.8[a]	91

Supplementary Table 3: Kinetic characterization of BH_{MeHis}1.0, BH_{MeHis}1.8 and BH_{MeHis}1.8 MeHis23His. Kinetic constants derived from global fitting of the combined V_0 vs [**1**] and V_0 vs [**2**] steady state kinetic data (Supplementary Figure 5) using a random order binding model. Saturating conditions for either **1** or **2** were not achieved for BH_{MeHis}1.0. K_M values quoted are the apparent Michaelis constants. N.D. = not determined. Source Data are provided as a Source Data file.

**Variant**	**k_{cat} (min^{-1})**	**K_M(1) (μM)**	**K_M(2) (μM)**
BH_{MeHis}1.0	N.D.	N.D.	N.D.
BH_{MeHis}1.8	4.50 ± 0.19	12020 ± 1034	323 ± 24
BH_{MeHis}1.8 MeHis23His	1.13 ± 0.05	12190 ± 1079	293 ± 22

Supplementary Table 4: Conversions of MBH reactions catalysed by BH_{MeHis}1.0, BH_{MeHis}1.8 and selected variants. MBH reaction conversions of BH_{MeHis}1.0, BH_{MeHis}1.8 and selected variants after 2 h using **1** (15 mM), **2** (2 mM) in PBS pH 7.0 with 3% MeCN as cosolvent.

[a] Conversion for preparative-scale biotransformation performed using **1** (50 mM), **2** (10 mM) in PBS pH 7.0 with 20% (v/v) DMSO as cosolvent.

Variant	Catalyst loading (mol%)	Time (h)	Conversion (%)
BH_{MeHis}1.0	0.1	2	<0.5
BH_{MeHis}1.8	0.1	2	26
BH_{MeHis}1.8 MeHis23Ala	0.1	2	<0.5
BH_{MeHis}1.8 MeHis23His	0.1	2	6.6
BH_{MeHis}1.8 Glu26Gln	0.1	2	1.3
BH_{MeHis}1.8 Glu26Ala	0.1	2	<0.5
BH_{MeHis}1.8 Trp42Phe	0.1	2	11
BH_{MeHis}1.8 [a]	0.1	13	96
N-methylimidazole	100	24	2.2
Imidazole	100	24	2.5

Supplementary Table 5: Effect of cosolvent on BH_{MeHis}1.8 activity. Reaction conversions of BH_{MeHis}1.8 with varying cosolvent loadings from 3% to 50% (v/v) of either MeCN or DMSO. Reactions performed using **1** (15 mM), **2** (1.5 mM) in PBS pH 7.0 with 3 µM BH_{MeHis}1.8 and 2 h incubation at 30 °C. Standard deviation of measurements made in triplicate. N.D. = not detectable. Source Data are provided as a Source Data file.

Cosolvent	Conversion (%)	S.D.
3% MeCN	40.19	0.36
5% MeCN	38.15	0.75
10% MeCN	29.94	0.78
15% MeCN	15.81	0.23
20% MeCN	5.74	0.34
30% MeCN	0.27	0.04
40% MeCN	N.D.	N.D.
50% MeCN	N.D.	N.D.
3% DMSO	45.41	0.06
5% DMSO	46.52	0.16
10% DMSO	50.04	0.13
15% DMSO	50.24	0.96
20% DMSO	50.04	0.62
30% DMSO	41.08	1.37
40% DMSO	12.90	0.44
50% DMSO	N.D.	N.D.

Supplementary Table 6: Reaction conditions for the substate scope to synthesise 3 and 4a-k. All reactions were performed in triplicate with 1mol% $BH_{MeHis}1.8$ (unless otherwise stated) at 30˚C in PBS pH 7.0 with 20% (v/v) DMSO as cosolvent. Conversion to product stated. Source Data are provided as a Source Data file.

Product	Alkene Conc. (mM)	Aldehyde Conc. (mM)	Time (h)	Mean % Conv. (±s.d.)
**3**[a]	50	10	2	97 ± 0.2
**4a**	50	10	24	99.7 ± 0.01
**4b**	50	10	24	92 ± 0.1
**4c**	100	10	4	66 ± 0.3
**4d**	100	10	4	72 ± 0.1
**4e**	100	10	7	91 ± 0.6
**4f**[b]	100	10	24	62 ± 1.1
**4g**	100	10	8	5 ± 0.09
**4h**	100	10	2	98 ± 0.9
**4i**	100	10	7	95 ± 0.1
**4j**	100	10	4	88 ± 1.5
**4k + 4l**	50	10	24	98 ± 0.2

[a] Reaction ran with 0.5mol% $BH_{MeHis}1.8$

[b] Extinction coefficients of 3276 and 724 $mM^{-1}cm^{-1}$ calculated for aldehyde and product, respectively, at 254nm.

Supplementary Table 7: Data collection and refinement statistics.
[a]Values in parentheses are for highest resolution shell. [b]R-free was calculated using ~5% of the data separate from the rest.

	$BH_{MeHis}1.0$	$BH_{MeHis}1.8$
PDB ascension number	8BP1	8BP0
Wavelength (Å)	0.9762	0.9763
Resolution range	61.5 - 1.72 (1.782 - 1.72)[a]	38.15 - 2.621 (2.714 - 2.621)[a]
Space group	P 31 2 1	P 21 21 21
Unit cell dimensions<br>a, b, c, (Å)<br>α, β, γ (°)	71.01, 71.01, 120.3<br>90, 90, 120	35.46, 42.43, 152.5<br>90, 90, 90
Total reflections	765674 (70921)	193638 (18122)
Unique reflections	37997 (3733)	7422 (715)
Multiplicity	20.2 (19.0)	26.1 (25.3)
Completeness (%)	99.94 (99.71)	99.81 (99.58)
Mean I/sigma(I)	20.45 (1.00)	9.87 (2.25)
Wilson B-factor	30.52	33.11
R-merge	0.08128 (1.691)	0.2535 (0.8861)
R-meas	0.08336 (1.736)	0.2585 (0.9041)
R-pim	0.01838 (0.3912)	0.04998 (0.1782)
$CC_{1/2}$	1 (0.709)	0.996 (0.964)
CC*	1 (0.911)	0.999 (0.991)
Reflections used in refinement	37987 (3733)	7411 (713)
Reflections used for R-free	1865 (170)	400 (38)
R-work	0.1811 (0.2491)	0.1902 (0.2495)
R-free[b]	0.2021 (0.2687)	0.2419 (0.3119)
CC (work)	0.960 (0.826)	0.958 (0.942)
CC (free)	0.959 (0.805)	0.912 (0.706)
Number of non-hydrogen atoms	2170	1962
macromolecules	1948	1867
ligands	22	18
solvent	200	77
Protein residues	231	230
RMS(bonds)	0.004	0.002
RMS (angles)	0.74	0.45
Ramachandran favoured (%)	98.67	95.11
Ramachandran allowed (%)	1.33	4.89
Ramachandran outliers (%)	0	0
Rotamer outliers (%)	0	3.08
Clashscore	3.24	2.1
Average B-factor	38.59	36.87

Supplementary Table 8: Kinetic isotope effect (KIE) and solvent kinetic isotope (SKIE) effects for BH_{MeHis}1.8 and selected variants. Reactions were performed with the relevant enzyme (1 µM BH_{MeHis}1.8, 3 µM BH_{MeHis}1.8 MeHis23His and 10 µM BH_{MeHis}1.8 Glu26Gln) using **1** or **S2** (25 mM), **2** (2 mM) in both deuterated and non-deuterated PBS buffer at pH 7.0 with 3% (v/v) MeCN as cosolvent. KIE and SKIE values calculated from reactions performed in triplicate. Source Data are provided as a Source Data file.

Variant	KIE (H_2O)	KIE (D_2O)	SKIE (1)	SKIE (S2)
BH_{MeHis}1.8	1.7	1.7	0.9	0.9
BH_{MeHis}1.8 Glu26Gln	4.0	4.2	0.6	0.6
BH_{MeHis}1.8 MeHis23His	1.9	1.5	0.6	0.5

Supplementary Table 9: Experimental and calculated mass values of enzymes in this study.

Variant	Expected Mass	Observed Mass
$BH_{MeHis}1.0$ (BH32.8 His23MeHis)	27593.73	27593.4
$BH_{MeHis}1.1$	27567.60	27567.4
$BH_{MeHis}1.2$	27624.66	27624.4
$BH_{MeHis}1.3$	27522.52	27522.2
$BH_{MeHis}1.4$	27546.59	27546.2
$BH_{MeHis}1.5$	27387.36	27387.0
$BH_{MeHis}1.6$	27520.51	27520.2
$BH_{MeHis}1.7$	27508.55	27508.4
$BH_{MeHis}1.8$	27515.54	27515.5
$BH_{MeHis}1.8$ MeHis23His	27501.51	27501.2
$BH_{MeHis}1.8$ MeHis23Ala	27435.44	27435.3
$BH_{MeHis}1.8$ Glu26Ala	27457.50	27457.3
$BH_{MeHis}1.8$ Glu26Gln	27514.52	27514.2
$BH_{MeHis}1.8$ (strep tag)	27732.84	27732.4
$BH_{MeHis}1.8$ MeHis23His (strep tag)	27718.81	27718.3
BH32.8 ($BH_{MeHis}1.0$ MeHis23His)	27579.70	27579.2
$BH_{MeHis}1.1$ MeHis23His	27553.57	27553.4
$BH_{MeHis}1.2$ MeHis23His	27610.63	27610.4
$BH_{MeHis}1.3$ MeHis23His	27508.49	27508.2
$BH_{MeHis}1.4$ MeHis23His	27532.56	27532.2
$BH_{MeHis}1.5$ MeHis23His	27373.33	27373.2
$BH_{MeHis}1.6$ MeHis23His	27506.48	27506.4
$BH_{MeHis}1.7$ MeHis23His	27494.52	27494.4
BH32 His23MeHis	27648.58	27648.6
BH32.6 His23MeHis	27525.55	27545.4
BH32.9 His23MeHis	27716.77	27716.6
BH32.12 His23MeHis	27529.59	27529.4

Supplementary Table 10: Table of primers used in this study

Name	Primer Sequence
BH32-BH32.6_His23MeHis_F	GGCGCTGCTAAATCC**TAG**CTGAAAATTATGGAGGAAGTG
BH32-BH32.6_His23_R	GGATTTAGCAGCGCC
BH32.8_His23MeHis_F	GGCGCTTATAAAGTG**TAG**CTGAAAATTATGGAGGAAGTG
BH32.8_His23_R	CACTTTATAAGCGCCTTC
BH32.9-BH32.14_His23MeHis_F	GGCGCTTATAAAGTG**TAG**TTTAAAATTATGGAGG
BH32.9-BH32.14_His23_R	CACTTTATAAGTGCCTTCAA
BH$_{\text{MeHis}}$1.8_MeHis23Ala_F	GGCGCTTATAAAATT**GCG**GTGAAAGAGATGGAGGAAG
BH$_{\text{MeHis}}$1.8_MeHis23His_F	GGCGCTTATAAAATT**CAC**GTGAAAGAGATGGAGGAAG
BH$_{\text{MeHis}}$1.8_MeHis23_R	AATTTTATAAGCGCCTTCAACG
BH$_{\text{MeHus}}$1.8_Glu26Ala_F	AAAATTTAGGTGAAA**GCG**ATGGAGGAAGTGCTGG
BH$_{\text{MeHus}}$1.8_Glu26Gln_F	AAAATTTAGGTGAAA**CAG**ATGGAGGAAGTGCTGG
BH$_{\text{MeHus}}$1.8_Glu26_R	TTTCACCTAAATTTTATAAGCGCCTTC
BH$_{\text{MeHus}}$1.8_Trp42Phe_F	AACCCGCGAACCCTG**TTT**GACGAATTTGATAAACTGTTTAAGG
BH$_{\text{MeHus}}$1.8_Trp42_R	CAGGGTTCGCGG
BH$_{\text{MeHis}}$1.1-1.2_MeHis23His_F	GGCGCTTATAAAGTG**CAC**CTGAAAGAGATGGAGGAAG
BH$_{\text{MeHis}}$1.1-1.2_MeHis23_R	CACTTTATAAGCGCCTTCA
BH$_{\text{MeHis}}$1.3-1.5_MeHis23His_F	GGCGCTTATAAAGTG**CAC**GTGAAAGAGATGGAGGAAG
BH$_{\text{MeHis}}$1.3-1.5_MeHis_R	CACTTTATAAGCGCCTTCA
BH$_{\text{MeHis}}$1.6-1.7_MeHis23His_F	GGCGCTTATAAAATT**CAC**GTGAAAGAGATGGAGGAAG
BH$_{\text{MeHis}}$1.6-1.7_MeHis23_R	AATTTTATAAGCGCCTTCAACG

Supplementary Table 11: Table of primers used for library generation

**Flanking Primers**	
NDE_F	catgcatCATATGATTCGTGCGGTA
XHO_R	atgcatgcCTCGAGAGAGCCCTG
**Round 1**	
L10NNK_F	GTATTCTTTGATAGC**NNK**GGTACTCTGATTAGCGT
L10_R	GCTATCAAAGAATACCGC
A19NNK_F	ATTAGCGTTGAAGGC**NNK**TATAAAGTGTAGCTGAAAATTATGG
A19_R	GCCTTCAACGCTAATC
Y20NNK_F	AGCGTTGAAGGCGCT**NNK**AAAGTGTAGCTGAAAATTATGG
Y20_R	AGCGCCTTCAACG
V22NNK_F	GAAGGCGCTTATAAA**NNK**TAGCTGAAAATTATGGAGGA
V22_R	TTTATAAGCGCCTTCAAC
L24NNK_F	GCTTATAAAGTGTAG**NNK**AAAATTATGGAGGAAGTGC
L24_R	CTACACTTTATAAGCGCC
I26NNK_F	AAAGTGTAGCTGAAA**NNK**ATGGAGGAAGTGCTG
I26_R	TTTCAGCTACACTTTATAAGC
M27NNK_F	GTGTAGCTGAAAATT**NNK**GAGGAAGTGCTGGGT
M27_R	AATTTTCAGCTACACTTTATAAG
L42NNK_F	AACCCGAAAACCCTG**NNK**GACGAATACGAGAAACTG
L42_R	CAGGGTTTTCGGGTT
Y45NNK_F	ACCCTGCTGGACGAA**NNK**GAGAAACTGGCTCGC
Y45_R	TTCGTCCAGCAGG
E46NNK_F	CTGCTGGACGAATAC**NNK**AAACTGGCTCGCG
E46_R	GTATTCGTCCAGCAGG
Y56NNK_F	GAAGCGTTCTCTAAC**NNK**GCGGGCAAACCG
Y56_R	GTTAGAGAACGCTTCGC
L64NNK_F	AAACCGTATCGTCCG**NNK**CGTGATATCCTGGAAGA
L64_R	CGGACGATACGGTTT
E70NNK_F	CGTGATATCCTGGAA**NNK**GTAATGCGTAAACTGGC
E70_R	TTCCAGGATATCACGC

D125NNK_F	GTGATCCTGAATAGG**NNK**ACCGAGCCGGC
D125_R	CCTATTCAGGATCACGC
P128NNK_F	AATAGGGATACCGAG**NNK**GCCACGGCATTCC
P128_R	CTCGGTATCCCTATTCAG
A129NNK_F	AGGGATACCGAGCCG**NNK**ACGGCATTCCTGGA
A129_R	CGGCTCGGTATCC
F132NNK_F	GAGCCGGCCACGGCA**NNK**CTGGACGCACTGG
F132_R	TGCCGTGGCCG
F154NNK_F	GAAGAAGCTGGTTTC**NNK**AAACCGCACCCAC
F154_R	GAAACCAGCTTCTTCAG
Y177NNK_F	GGCGAGAAAGCAGTG**NNK**GTTGGTGACAACCCG
Y177_R	CACTGCTTTCTCGCC
D180NNK_F	GCAGTGTACGTTGGT**NNK**AACCCGGTCAAAGAC
D180_R	ACCAACGTACACTGC
**Round 2**	
L42x_Y45x_1	AACCCGAAAACCCTG**NDT**GACGAA**NDT**GAGAAACTGGCTCGC
L42x_Y45x_2	AACCCGAAAACCCTG**VHG**GACGAA**VHG**GAGAAACTGGCTCGC
L42x_Y45x_3	AACCCGAAAACCCTG**NDT**GACGAA**VHG**GAGAAACTGGCTCGC
L42x_Y45x_4	AACCCGAAAACCCTG**VHG**GACGAA**NDT**GAGAAACTGGCTCGC
L42x_Y45x_5	AACCCGAAAACCCTG**NDT**GACGAA**TGG**GAGAAACTGGCTCGC
L42x_Y45x_6	AACCCGAAAACCCTG**TGG**GACGAA**NDT**GAGAAACTGGCTCGC
L42x_Y45x_7	AACCCGAAAACCCTG**VHG**GACGAA**TGG**GAGAAACTGGCTCGC
L42x_Y45x_8	AACCCGAAAACCCTG**TGG**GACGAA**VHG**GAGAAACTGGCTCGC
L42x_Y45x_9	AACCCGAAAACCCTG**TGG**GACGAA**TGG**GAGAAACTGGCTCGC
L42x_Y45x_R	CAGGGTTTTCGGGTT
**Round 3**	
NDE_S9NNK_F	gagatatacatATGATTCGTGCGGTATTCTTTGAT**NNK**CTGGGTACTCTGATTAGC
I14NNK_F	AGCCTGGGTACTCTG**NNK**AGCGTTGAAGGCGCT
I14_R	CAGAGTACCCAGGCT
A19NNK_F	ATTAGCGTTGAAGGC**NNK**TATAAAGTGTAGCTGAAAGAG
A19_R	GCCTTCAACGCTAATC
Y20NNK_F	AGCGTTGAAGGCGCT**NNK**AAAGTGTAGCTGAAAGAGA

Y20_R	AGCGCCTTCAACG
V22NNK_F	GAAGGCGCTTATAAA**NNK**TAGCTGAAAGAGATGGAG
V22_R	TTTATAAGCGCCTTCAAC
L24NNK_F	GCTTATAAAGTGTAG**NNK**AAAGAGATGGAGGAAGTG
L24_R	CTACACTTTATAAGCGCC
M27NNK_F	GTGTAGCTGAAAGAG**NNK**GAGGAAGTGCTGGG
M27_R	CTCTTTCAGCTACACTTT
A49NNK_F	GAATACGAGAAACTG**NNK**CGCGAAGCGTTCT
A49_R	CAGTTTCTCGTATTCGTC
F53NNK_F	CTGGCTCGCGAAGCG**NNK**TCTAACTATGCGGGC
F53_R	CGCTTCGCGAGCC
L87NNK_F	AAATACCCTGAAAAC**NNK**TGGGAAATCTCCCTG
L87_R	GTTTTCAGGGTATTTGAAAC
W88NNK_F	TACCCTGAAAACTTG**NNK**GAAATCTCCCTGCGT
W88_R	CAAGTTTTCAGGGTATTTGA
S91NNK_F	AACTTGTGGGAAATC**NNK**CTGCGTATGGCGC
S91_R	GATTTCCCACAAGTTTTCA
L92NNK_F	TTGTGGGAAATCTCC**NNK**CGTATGGCGCAACGC
L92_R	GGAGATTTCCCACAAGT
A95NNK_F	ATCTCCCTGCGTATG**NNK**CAACGCTACGGCGAG
A95_R	CATACGCAGGGAGAT
N123NNK_F	GTTGGCGTGATCCTG**NNK**AGGGATACCGAGCC
N123_R	CAGGATCACGCCAAC
D125NNK_F	GTGATCCTGAATAGG**NNK**ACCGAGCCGGC
D125_R	CCTATTCAGGATCACGC
A131NNK_F	ACCGAGCCGGCCACG**NNK**TTCCTGGACGCAC
A131_R	CGTGGCCGGCTC
F132NNK_F	GAGCCGGCCACGGCA**NNK**CTGGACGCACTGG
F132_R	TGCCGTGGCCG
K174NNK_F	GGCGTTAAAGGCGAG**NNK**GCAGTGTACGTTGGT
K174_R	CTCGCCTTTAACGCC
**Round 5**	

G32NNK_F	ATGGAGGAAGTGCTG**NNK**GACTATCCGCTGAACC
G32_R	CAGCACTTCCTCCAT
E46NNK_F	CTGTGGGACGAATTT**NNK**AAACTGGCTCGCGAA
E46_R	AAATTCGTCCCACAGG
L48NNK_F	GACGAATTTGAGAAA**NNK**GCTCGCGAAGCG
L48_R	TTTCTCAAATTCGTCCCA
E51NNK_F	GAGAAACTGGCTCGC**NNK**GCGTTCTCTAACTATGCG
E51_R	GCGAGCCAGTTTCTC
D66NNK_F	TATCGTCCGGCGCGT**NNK**ATCCTGGAAGAAGTAATGC
D66_R	ACGCGCCGGAC
V71NNK_F	GATATCCTGGAAGAA**NNK**ATGCGTAAACTGGCG
V71_R	TTCTTCCAGGATATCACG
K74NNK_F	GAAGAAGTAATGCGT**NNK**CTGGCGGAAAAGTACG
K74_R	ACGCATTACTTCTTCCA
E85NNK_F	GGTTTCAAATACCCT**NNK**AACTTGCAGGAAATCGG
E85_R	AGGGTATTTGAAACCGT
Q96NNK_F	GGCCTGCGTATGGCG**NNK**CGCTACGGCGAGC
Q96_R	CGCCATACGCAGG
R124NNK_F	GGCGTGATCCTGAAT**NNK**GATACCGAGCCGGC
R124_R	ATTCAGGATCACGCC
K139NNK_F	GACGCACTGGGCATC**NNK**GACCTGTTCGATTCCA
K139_R	GATGCCCAGTGCGT
T146NNK_F	CTGTTCGATTCCATC**NNK**ACGTCTGAAGAAGCT
T146_R	GATGGAATCGAACAGGT
F161NNK_F	CCGCACCCACGCATC**NNK**GAACTGGCTCTGAAGA
F161_R	GATGCGTGGGTGC
E173NNK_F	GCCGGCGTTAAAGGC**NNK**AAAGCAGTGTACGTTGG
E173_R	GCCTTTAACGCCGG
Y177NNK_F	GGCGAGAAAGCAGTG**NNK**GTTGGTGACAACCCG
Y177_R	CACTGCTTTCTCGCC
V178NNK_F	GAGAAAGCAGTGTAC**NNK**GGTGACAACCCGGTC
V178_R	GTACACTGCTTTCTCGC

K184NNK_F	GGTGACAACCCGGTC**NNK**GACGCGGGTGGTT
K185_R	GACCGGGTTGTCACC
D185NNK_F	GACAACCCGGTCAAA**NNK**GCGGGTGGTTCTAAG
D185_R	TTTGACCGGGTTGTC
K202NNK_F	ATCCTGCTGGATCGT**NNK**GGTGAGAAACGTGAATTC
K202_R	ACGATCCAGCAGGAT
I215NNK_F	GATAAGGCGGACTTT**NNK**GTCTCCGACCTGC
I215_R	AAAGTCCGCCTTATCC
S217NNK_F	GCGGACTTTATCGTC**NNK**GACCTGCGCGAAGTT
S217_R	GACGATAAAGTCCGC
I223NNK_F	GACCTGCGCGAAGTT**NNK**AAGATTGTTGACGAACTGA
I223_R	AACTTCGCGCAGGTC
**Round 6**	
V22NNK_F	GAAGGCGCTTATAAA**NNK**TAGGTGAAAGAGATGGAGG
V22_R	TTTATAAGCGCCTTCAAC
M27NNK_F	GTGTAGGTGAAAGAG**NNK**GAGGAAGTGCTGGG
M27_R	CTCTTTCACCTACACTTTAT
Y34NNK_F	GAAGTGCTGGGTGAC**NNK**CCGCTGAACCCG
Y34_R	GTCACCCAGCACTTC
K39NNK_F	TATCCGCTGAACCCG**NNK**ACCCTGTGGGACG
K39_R	CGGGTTCAGCGGATA
L41NNK_F	CTGAACCCGAAAACCNNKTGGGACGAATTTGAGAA
L41_R	GGTTTTCGGGTTCAGC
E46NNK_F	CTGTGGGACGAATTT**NNK**AAACTGGCTCGCGAA
E46_R	AAATTCGTCCCACAGG
F53NNK_F	CTGGCTCGCGAAGCG**NNK**TCTAACTATGCGGGCA
F53_R	CGCTTCGCGAGC
Y56NNK_F	GAAGCGTTCTCTAAC**NNK**GCGGGCAAACCGTAT
Y56_R	GTTAGAGAACGCTTCGC
D66NNK_F	TATCGTCCGGCGCGT**NNK**ATCCTGGAAGAAGTAATGC
D66_R	ACGCGCCGGAC
E85NNK_F	GGTTTCAAATACCCT**NNK**AACTTGCAGGAAATCGG

E85_R	AGGGTATTTGAAACCGT
L92NNK_F	TTGCAGGAAATCGGC**NNK**CGTATGGCGCGAC
L92_R	GCCGATTTCCTGCAA
A95NNK_F	ATCGGCCTGCGTATG**NNK**CGACGCTACGGC
A95_R	CATACGCAGGCCGAT
K110NNK_F	GTGGTGGAAGTACTG**NNK**TCTCTGAAAGGTAAATATCACG
K110_R	CAGTACTTCCACCACTT
Y116NNK_F	TCTCTGAAAGGTAAA**NNK**CACGTTGGCGTGATC
Y116_R	TTTACCTTTCAGAGATTTCAGT
L122NNK_F	CACGTTGGCGTGATC**NNK**AATTGGGATACCGAGC
L122_R	GATCACGCCAACGTG
D125NNK_F	GTGATCCTGAATTGG**NNK**ACCGAGCCGGC
D125_R	CCAATTCAGGATCACGC
P128NNK_F	AATTGGGATACCGAG**NNK**GCCACGGCATTCCTG
P128_R	CTCGGTATCCCAATTCA
F132NNK_F	GAGCCGGCCACGGCA**NNK**CTGGACGCACTGGG
F132_R	TGCCGTGGCCG
F154NNK_F	GAAGAAGCTGGTTTC**NNK**AAACCGCACCCACG
F154_R	GAAACCAGCTTCTTCAG
K155NNK_F	GAAGCTGGTTTCTTT**NNK**CCGCACCCACGC
K155_R	AAAGAAACCAGCTTCTTCA
E173NNK_F	GCCGGCGTTAAAGGC**NNK**AAAGCAGTGTACGTTGG
E173_R	GCCTTTAACGCCGG
K174NNK_F	GGCGTTAAAGGCGAG**NNK**GCAGTGTACGTTGGT
K174_R	CTCGCCTTTAACGCC
A175NNK_F	GTTAAAGGCGAGAAA**NNK**GTGTACGTTGGTGACA
A175_R	TTTCTCGCCTTTAACGC
Y177NNK_F	GGCGAGAAAGCAGTG**NNK**GTTGGTGACAACCCG
Y177_R	CACTGCTTTCTCGCC
V178NNK_F	GAGAAAGCAGTGTAC**NNK**GGTGACAACCCGGTC
V178_R	GTACACTGCTTTCTCGC
E207NNK_F	AAAGGTGAGAAACGT**NNK**TTCTGGGATAAGGCGG

E207_R	ACGTTTCTCACCTTTACG
I215NNK_F	GATAAGGCGGACTTT**NNK**GTCTCCGACCTGCG
I215_R	AAAGTCCGCCTTATCC
A212NNK_F	GAATTCTGGGATAAG**NNK**GACTTTATCGTCTCCGAC
A212_R	CTTATCCCAGAATTCACGT
**Round 7**	
NDE_S9NNK_F	gagatatacatATGATTCGTGCGGTATTCTTTGAT**NNK**CTGGGTACTCTGATTAGC
NDE_L10NNK_F	gagatatacatATGATTCGTGCGGTATTCTTTGATAGC**NNK**GGTACTCTGATTAGCGT
V22NNK_F	GAAGGCGCTTATAAA**NNK**TAGGTGAAAGAGATGGAG
V22_R	TTTATAAGCGCCTTCAAC
Y34NNK_F	GAAGTGCTGGGTGAC**NNK**CCGCTGAACCCG
Y34_R	GTCACCCAGCACTTC
K39NNK_F	TATCCGCTGAACCCG**NNK**ACCCTGTGGGACG
K39_R	CGGGTTCAGCGGATA
L41NNK_F	CTGAACCCGAAAACC**NNK**TGGGACGAATTTGATAAACT
L41_R	GGTTTTCGGGTTCAGC
W42NNK_F	AACCCGAAAACCCTG**NNK**GACGAATTTGATAAACTGGC
W42_R	CAGGGTTTTCGGGTT
A49NNK_F	GAATTTGATAAACTG**NNK**CGCGAAGCGTTCTCT
A49_R	CAGTTTATCAAATTCGTCCC
F53NNK_F	CTGGCTCGCGAAGCG**NNK**TCTAACTATGCGGGC
F53_R	CGCTTCGCGAGCC
Y56NNK_F	GAAGCGTTCTCTAAC**NNK**GCGGGCAAACCGTAT
Y56_R	GTTAGAGAACGCTTCGC
D66NNK_F	TATCGTCCGGCGCGT**NNK**ATCCTGGAAGAAGTAATGC
D66_R	ACGCGCCGGAC
E85NNK_F	GGTTTCAAATACCCT**NNK**AACTTGCAGGAAATCGG
E85_R	AGGGTATTTGAAACCGT
G91NNK_F	AACTTGCAGGAAATC**NNK**GTGCGTATGGCGC
G91_R	GATTTCCTGCAAGTTTTCAG
A95NNK_F	ATCGGCGTGCGTATG**NNK**CGACGCTACGGCGAG
A95_R	CATACGCACGCCGAT

L122NNK_F	CACGTTGGCGTGATC**NNK**AATTGGGATACCGAGAAT
L122_R	GATCACGCCAACGTG
D125NNK_F	GTGATCCTGAATAGG**NNK**ACCGAGAATGCCAC
D125_R	CCAATTCAGGATCACGC
E127NNK_F	CTGAATTGGGATACC**NNK**AATGCCACGGCATTC
E127_R	GGTATCCCAATTCAGGAT
N128NNK_F	AATTGGGATACCGAG**NNK**GCCACGGCATTCCTG
N128_R	CTCGGTATCCCAATTCA
F154NNK_F	GAAGAAGCTGGTTTC**NNK**AAACCGCACCCACG
F154_R	GAAACCAGCTTCTTCAG
K155NNK_F	GAAGCTGGTTTCTTT**NNK**CCGCACCCACGC
K155_R	AAAGAAACCAGCTTCTTCA
Y177NNK_F	GGCGAGAAAGCAGTG**NNK**GTTGGTGACAACCCG
Y177_R	CACTGCTTTCTCGCC
I215NNK_F	GATAAGGCGGACTTT**NNK**GTCTCCGACCTGCG
I215_R	AAAGTCCGCCTTATCC
**Round 9**	
NDE_S15NNK_F	gagatatacatATGATTCGTGCGGTATTCTTTGATAGCCTGGGTACTCTGATT**NNK**GTTGAAGGCGC TTATAAAA
E26NNK_F	AAAATTTAGGTGAAA**NNK**ATGGAGGAAGTGCTG
E26_R	TTTCACCTAAATTTTATAAGCGCC
M27NNK_F	ATTTAGGTGAAAGAG**NNK**GAGGAAGTGCTGGGT
M27_R	CTCTTTCACCTAAATTTTATAAGC
Y34NNK_F	GAAGTGCTGGGTGAC**NNK**CCGCTGAACCCGAAA
Y34_R	GTCACCCAGCACTTC
K39NNK_F	TATCCGCTGAACCCG**NNK**ACCCTGTGGGACGAA
K39_R	CGGGTTCAGCGGATA
D43NNK_F	CCGAAAACCCTGTGG**NNK**GAATTTGATAAACTGTTTCGCG
D43_R	CCACAGGGTTTTCGG
R62NNK_F	GCGGGCAAACCGTAT**NNK**CCGGCGCGTGATATC
R62_R	ATACGGTTTGCCCGC
D66NNK_F	TATCGTCCGGCGCGT**NNK**ATCCTGGAAGAAGTAATGC

D66_R	ACGCGCCGGAC
E100NNK_F	GCGCGACGCTACGGC**NNK**CTGTACCCGGAAGTG
E100_R	GCCGTAGCGTCG
L112NNK_F	GAAGTACTGAAATCT**NNK**AAAGGTAAATATCACGTTGGC
L112_R	AGATTTCAGTACTTCCACC
K115NNK_F	AAATCTCTGAAAGGT**NNK**TATCACGTTGGCGTG
K115_R	ACCTTTCAGAGATTTCAGT
L122NNK_F	CACGTTGGCGTGATC**NNK**AATTGGGATACCGAGAAT
L122_R	GATCACGCCAACGTG
N123NNK_F	GTTGGCGTGATCCTG**NNK**TGGGATACCGAGAATG
N123_R	CAGGATCACGCCAAC
D125NNK_F	GTGATCCTGAATTGG**NNK**ACCGAGAATGCCACG
D125_R	CCAATTCAGGATCACGC
L165NNK_F	ATCCTCGAACTGGCT**NNK**AAGAAAGCCGGCGTT
L165_R	AGCCAGTTCGAGGAT
E173NNK_F	GCCGGCGTTAAAGGC**NNK**AAAGCAGTGTACGTTGG
E173_R	GCCTTTAACGCCGG
V176NNK_F	AAAGGCGAGAAAGCA**NNK**TACGTTGGTGACAACC
V176_R	TGCTTTCTCGCCTTT
S189NNK_F	AAAGACGCGGGTGGT**NNK**AAGAACCTGGGTATGAC
S189_R	ACCACCCGCGTCT
N191NNK_F	GCGGGTGGTTCTAAG**NNK**CTGGGTATGACTAGCAT
N191_R	CTTAGAACCACCCGC
G193NNK_F	GGTTCTAAGAACCTG**NNK**ATGACTAGCATCCTGCT
G193_R	CAGGTTCTTAGAACCACC
R220NNK_F	CGTGTCTCCGACCTG**NNK**GAAGTTATTAAGATTGTTGACGAA
R220_R	CAGGTCGGAGACACG
XHO_D227NNM_R	TGGTGGTGCTCGAGAGAGCCCTGACCGTTCAGTTC**MNN**AACAATCTTAATAACTTCGCG
**Round 10**	
A20NNK_F	AGCGTTGAAGGCGCT**NNK**AAAATTTAGGTGAAAGAGATGG
A20_R	AGCGCCTTCAACG

R50NNK_F	TTTGATAAACTGTTT**NNK**GAAGCGTTCTCTAACTATG
R50_R	AAACAGTTTATCAAATTCGTCC
E51NNK_F	GATAAACTGTTTCGC**NNK**GCGTTCTCTAACTATGCG
E51_R	GCGAAACAGTTTATCAAATTCG
Y56NNK_F	GAAGCGTTCTCTAAC**NNK**GCGGGCAAACCG
Y56_R	GTTAGAGAACGCTTCGC
L68NNK_F	CCGGCGCGTTTTATC**NNK**GAAGAAGTAATGCGTAAACT
L68_R	GATAAAACGCGCCGG
L87NNK_F	AAATACCCTGGAAAC**NNK**CAGGAAATCGGCGTG
L87_R	GTTTCCAGGGTATTTGAAAC
M94NNK_F	GAAATCGGCGTGCGT**NNK**GCGCGACGCTAC
M94_R	ACGCACGCCGATTTC
A95NNK_F	ATCGGCGTGCGTATG**NNK**CGACGCTACGGC
A95_R	CATACGCACGCCGAT
R97NNK_F	GTGCGTATGGCGCGA**NNK**TACGGCGGGCTGTA
R97_R	TCGCGCCATACGC
Y98NNK_F	CGTATGGCGCGACGC**NNK**GGCGGGCTGTAC
Y98_R	GCGTCGCGCCATA
G99NNK_F	ATGGCGCGACGCTAC**NNK**GGGCTGTACCCGG
G99_R	GTAGCGTCGCGC
L101NNK_F	CGACGCTACGGCGGG**NNK**TACCCGGAAGTGGTG
L101_R	CCCGCCGTAGCG
Y102NNK_F	CGCTACGGCGGGCTG**NNK**CCGGAAGTGGTGGAA
Y102_R	CAGCCCGCCGTAG
T126NNK_F	ATCCTGAATTGGGAT**NNK**GAGAATGCCACGGCA
T126_R	ATCCCAATTCAGGATCAC
A129NNK_F	TGGGATACCGAGAAT**NNK**ACGGCATTCCTGGAC
A129_R	ATTCTCGGTATCCCAATT
F132NNK_F	GAGAATGCCACGGCA**NNK**CTGGACGCACTGG
F132_R	TGCCGTGGCATTCTC
T146NNK_F	CTGTTCGATTCCATC**NNK**ACGTCTGAAGAAGCTG
T146_R	GATGGAATCGAACAGGT

K155NNK_F	GAAGCTGGTTTCGCT**NNK**CCGCACCCACGC
K155_R	AGCGAAACCAGCTTC
A175NNK_F	GTTAAAGGCGAGAAA**NNK**GTGTACGTTGGTGACAA
A175_R	TTTCTCGCCTTTAACGC
Y177NNK_F	GGCGAGAAAGCAGTG**NNK**GTTGGTGACAACCCG
Y177_R	CACTGCTTTCTCGCC
E207NNK_F	AAAGGTGAGAAACGT**NNK**TTCTGGGATAAGGCG
E207_R	ACGTTTCTCACCTTTACG
A212NNK_F	GAATTCTGGGATAAG**NNK**GACTTTCGTGTCTCCG
A212_R	CTTATCCCAGAATTCACGT

2. **DNA and protein sequence for the most active variant BH_{MeHis}1.8.**

BH_{MeHis}1.8:

ATGATTCGTGCGGTATTCTTTGATAGCCTGGGTACTCTGATTAGCGTTGAAGGCGCTTATAAAATTTA
GGTGAAAGAGATGGAGGAAGTGCTGGGTGACTATCCGCTGAACCCGCGAACCCTGTGGGACGAATTTG
ATAAACTGTTTAAGGAAGCGTTCTCTAACTATGCGGGCAAACCGTATCGTCCGGCGCGTTTTATCCTG
GAAGAAGTAATGCGTAAACTGGCGGAAAAGTACGGTTTCAAATACCCTGGAAACTTGCAGGAAATCGG
CGTGCGTATGGCGCGACGCTACGGCGGGCTGTACCCGGAAGTGGTGGAAGTACTGAAATCTCTGAAAG
GTAAATATCACGTTGGCGTGATCCTGAATTGGGATACCGAGAATGCCACGGCATTCCTGGACGCACTG
GGCATCAAAGACCTGTTCGATTCCATCACCACGTCTGAAGAAGCTGGTTTCGCTTATCCGCACCCACG
CATCCTCGAACTGGCTCTGAAGAAAGCCGGCGTTAAAGGCGAGAAAGCAGTGTACGTTGGTGACAACC
CGGTCAAAGACGCGGGTGGTTCTAAGAACCTGGGTATGACTAGCATCCTGCTGGATCGTAAAGGTGAG
AAACGTGAATTCTGGGATAAGGCGGACTTTCGTGTCTCCGACCTGCGCGAAGTTATTAAGATTGTTGA
CGAACTGAACGGTCAGGGCTCTCTCGAGCACCACCACCACCACCACTGA

MIRAVFFDSLGTLISVEGAYKI**(MHS)**VKEMEEVLGDYPLNPRTLWDEFDKLFKEAFSNYAGKPYRPA
RFILEEVMRKLAEKYGFKYPGNLQEIGVRMARRYGGLYPEVVEVLKSLKGKYHVGVILNWDTENATAF
LDALGIKDLFDSITTSEEAGFAYPHPRILELALKKAGVKGEKAVYVGDNPVKDAGGSKNLGMTSILLD
RKGEKREFWDKADFRVSDLREVIKIVDELNGQGSLEHHHHHH

MHS = MeHis

Supplementary References

1. Bjelic, S. et al. Computational design of enone-binding proteins with catalytic activity for the Morita-Baylis-Hillman reaction. *ACS Chem. Biol*. **8**, 749–757 (2013).
2. Crawshaw, R. et al. Engineering an efficient and enantioselective enzyme for the Morita–Baylis–Hillman reaction. *Nat. Chem*. **14**, 313–320 (2022).
3. Luo, S., Wang, P. G. & Cheng, J. P. Remarkable rate acceleration of imidazole-promoted Baylis-Hillman reaction involving cyclic enones in basic water solution. *J. Org. Chem*. **69**, 555–558 (2003).
